# CMTM6 inhibits tumor growth and reverses chemoresistance by preventing ubiquitination of p21 in hepatocellular carcinoma

**DOI:** 10.1038/s41419-022-04676-1

**Published:** 2022-03-19

**Authors:** Yue Huang, Yingqin Zhu, Jieying Yang, Qiuzhong Pan, Jingjing Zhao, Mengjia Song, Chaopin Yang, Yulong Han, Yan Tang, Qijing Wang, Jia He, Yongqiang Li, Junyi He, Hao Chen, Desheng Weng, Tong Xiang, Jian Chuan Xia

**Affiliations:** 1 Department of Biotherapy, Sun Yat-sen University Cancer Center, Guangzhou, P. R. China; 2 Collaborative Innovation Center for Cancer Medicine, State Key Laboratory of Oncology in South China, Sun Yat-sen University Cancer Center, Guangzhou, China; 3 Department of Pancreato-Biliary Surgery, First Affiliated Hospital, Sun Yat-Sen University, Guangzhou, China

**Keywords:** Cancer therapeutic resistance, Tumour biomarkers, Liver cancer

## Abstract

Hepatocellular carcinoma is one of the most common malignancies and has a poor prognosis. The ubiquitin-proteasome pathway is required for the degradation of most short-lived proteins. CMTM6 has been implicated in the progression of various tumors, but its biological function and the underlying molecular mechanisms in HCC are still unknown. In this study, we found that the expression of CMTM6 was significantly reduced in HCC and predicted better prognosis of HCC patients. Through in vitro and in vivo experiments, CMTM6 was shown to inhibit the proliferation of HCC cells by blocking the G1/S phase transition. Mechanistically, CMTM6 interacted with p21 and prevented its ubiquitination mediated by SCF^SKP2^, CRL4^CDT2^ and APC/C^CDC20^ in a cell-cycle–independent manner. As a result, CMTM6 stabilized p21 protein, leading to the inactivation of pRB/E2F pathway. Additionally, CMTM6 sensitized HCC cells to doxorubicin and cisplatin, positively correlated with better clinical outcomes of the transarterial chemoembolization (TACE) treatment for postoperative recurrence. Taken together, our study reports a novel mechanism by which p21 can be stabilized by CMTM6 and pinpoints a crucial role of the CMTM6-p21 axis in suppressing the progression of HCC and sensitizing patients with postoperative recurrence to TACE treatment.

## Introduction

Hepatocellular carcinoma (HCC) is one of the most common human malignancies worldwide, especially in developing countries [1–3]. HCC is commonly diagnosed only at intermediate (Barcelona Clinic Liver Cancer (BCLC) stage B) or advanced (BCLC stage C) tumor stages, where only palliative treatment options can be offered [4]. Transarterial chemoembolization (TACE) using a mixture of a chemotherapeutic agent (such as doxorubicin (Dox) or cisplatin (DDP)) and lipiodol the recommended standard treatment for intermediate stage HCC (BCLC stage B) [5]. However, due to frequent drug resistance, TACE showed limited effect on improving the survival of HCC. Therefore, research focusing on improving understanding of the molecular mechanisms that underlying HCC progression and drug resistance will have critical importance for the development of more effective HCC treatment.

CMTM6 belongs to the chemokine-like factor (CKLF) -like MARVEL transmembrane domain-containing family (CMTM), which consists of eight members, namely CMTM1-8 [6]. Previous researches on CMTM6 mainly focused on its function in antitumor immunity [7, 8]. However, there are conflicting reports regarding the roles of CMTM6 in tumorigenesis. On the one hand, it has been reported that CMTM6 promoted neck squamous cell carcinoma (NSCC) cells stemness, epithelial–mesenchymal transition (EMT) and T-cell dysfunction [9]. Also, high expression of CMTM6 is reported to be related to poor clinical outcomes of oral squamous cell carcinoma [10], gastric cancer [11] and gliomas [12]. On the other hand, CMTM6 was found to be associated with better survival in non-small cell lung cancer [13] and lung adenocarcinoma [14]. Peng et al. also confirmed that CMTM6 and PD-L1 co-expression is associated with an active immune microenvironment and a favorable prognosis in colorectal cancer [15]. However, the expression and biological characteristics of CMTM6 in HCC remain unclear.

The cyclin-dependent kinase (CDK) inhibitor p21 (also known as p21^WAF1/Cip1^ or *CDKN1A*) is a key negative regulator of cell cycle progression [16]. By binding to several CDK/cyclin complexes, p21 was found to induce cell cycle arrests at specific stages [16–18]. In addition, by binding to proliferating cell nuclear antigen (PCNA), p21 interferes with PCNA-dependent DNA polymerase activity, which is required for DNA replication and repair processes [19, 20]. Under normal conditions, p21 is an unstable protein with a relatively short half-life [21, 22]. Ubiquitin-proteasome pathway is a major mechanism for the regulation of p21 through its degradation [21]. Although the ubiquitin-proteasome degradation of p21 has been well established, the regulatory factors involved in the process remain to be further investigated.

In this study, we identified CMTM6 as a tumor suppressor in HCC that can inhibit the HCC cell proliferation by blocking G1/S transition. Importantly, we presented the first evidence that CMTM6 interacts with p21 and prevents its ubiquitin-dependent degradation, leading to inactivation of the phosphorylated RB (pRB)/E2F1 pathway. CMTM6 was also found to sensitize HCC cells to TACE treatment with Dox and DDP. Our study presents a novel mechanism that stabilizes p21 protein and reveals CMTM6 as a pivotal suppressor in HCC tumor progression and TACE-resistance.

## Results

### CMTM6 is downregulated in tumor tissues and its deficiency predicts poor prognosis of HCC patients

**Fig. 1:**
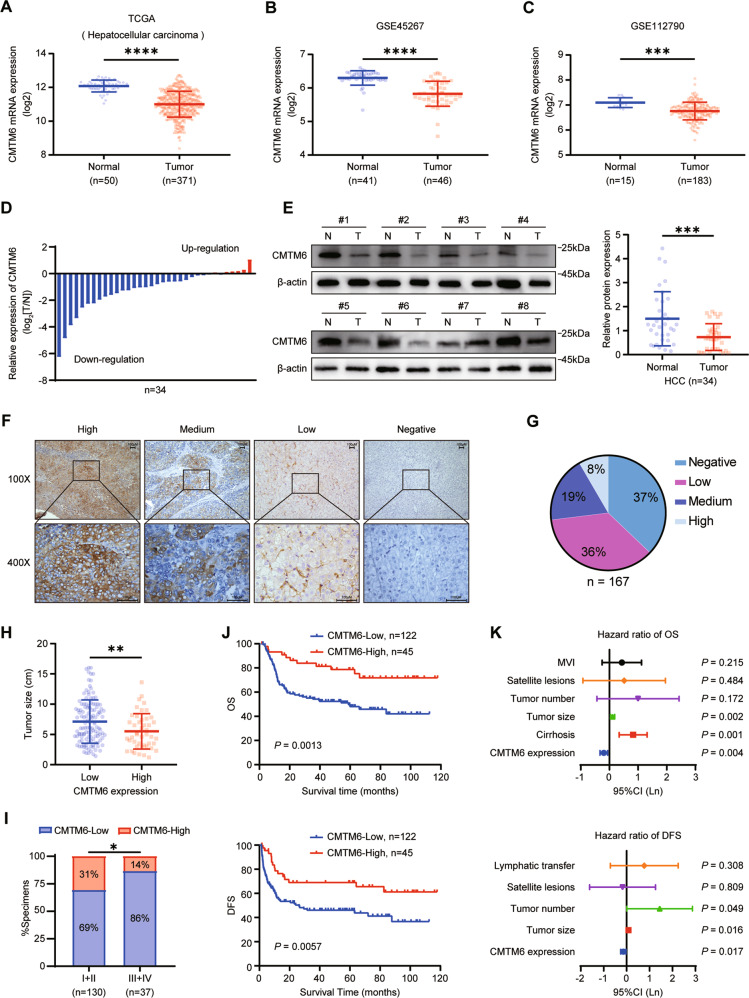
CMTM6 is downregulated in tumor tissues and its deficiency predicts poor prognosis in HCC. **A**–**C** CMTM6 mRNA levels of HCC tumor tissues (T) and normal liver tissues (N) as indicated in the liver hepatocellular carcinoma (LIHC) dataset from TCGA database (**A**) and the public GEO liver cancer datasets GSE45267 (**B**) and GSE112790 (**C**). **D** Results of the qPCR analysis of 34 pairs of HCC tumor tissues and adjacent normal liver tissues. **E** Representative images and statistical analysis of the Western blotting of 34 pairs of HCC tumor tissues and adjacent normal liver tissues. **F** Typical IHC images of CMTM6 expression status in HCC tissues. **G** The percentages of cases of four staining intensities. **H** Tumor size comparison according to CMTM6 expression status. **I** The percentages of CMTM6 expression status in early (TNM I + II) and advanced (TNM III + IV) HCC. **J** Kaplan–Meier analysis of OS (top) and DFS (bottom) according to CMTM6 expression status in 167 HCC patients. **K** Multivariate analysis of CMTM6 expression and clinical variables in HCC using a Cox-regression model. The error bars represent the mean ± SD. *P < 0.05; **P < 0.01; ***P < 0.001; ****P < 0.0001.

To determine the roles of CMTM6 in HCC progression, we first analyzed the expression of CMTM6 according to The Cancer Genome Atlas (TCGA) database and Gene Expression Omnibus (GEO) datasets (GSE45267 and GSE112790). CMTM6 expression was downregulated in liver cancer samples compared with that in normal liver tissue samples (Fig. 1A–C). qPCR and Western blot analysis in HCC tissues confirmed that CMTM6 mRNA and protein expression were downregulated in HCC tumor tissues compared with that in paired normal liver tissues (Fig. 1D, E). Likewise, human HCC cell lines (HepG2, Hep3B, HuH-7, SK-Hep-1) expressed lower CMTM6 levels than the normal liver cell line (L-02) (Fig. S1A, B). Furthermore, immunohistochemistry (IHC) analysis was performed in 167 archived HCC samples. The staining intensity (SI) levels of CMTM6 were divided into four groups: negative (0 ≤ SI < 3, 37%), low (3 ≤ SI < 5, 35%), medium (5 ≤ SI < 7, 19%), high (7 ≤ SI < 9, 9%) (Fig. 1F, G). Univariate analysis showed that CMTM6 expression was strongly associated with the tumor size (*P* = 0.027), TNM stage (*P* = 0.037), BCLC stage (*P* = 0.026) and postoperative recurrence (*P* = 0.022) (Fig. 1H, I, Table S3). The Kaplan–Meier analysis revealed that HCC patients with high CMTM6 expression had a significantly longer median overall survival (OS; 61.95 ± 34.42 vs. 38.65 ± 30.11, months) and disease-free survival (DFS; 52.90 ± 40.55 vs. 29.30 ± 31.66, months) than those with low CMTM6 expression (Fig. 1J). In addition, multivariate analysis revealed that the CMTM6 expression status was an independent indicator of both OS and DFS (Fig. 1K, Table S4 and S5). Taken together, these results indicated that CMTM6 might act as a tumor suppressor and be a useful prognostic biomarker in HCC.

### CMTM6 inhibits HCC tumor growth in vitro and in vivo

**Fig. 2:**
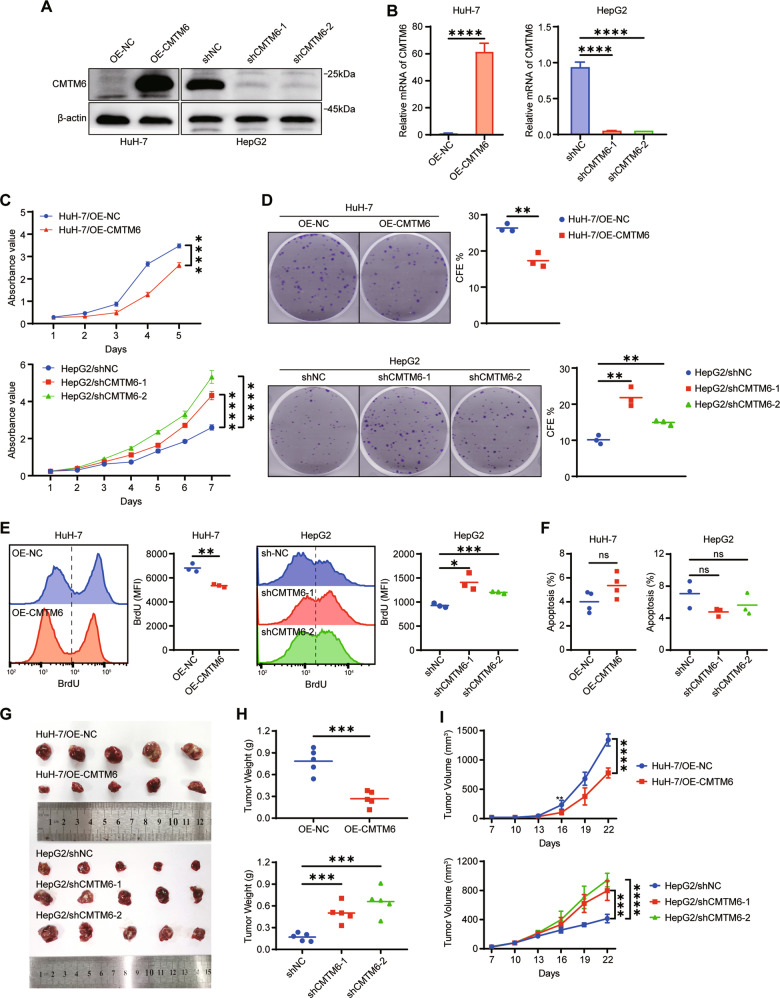
CMTM6 suppresses HCC tumor growth in vitro and in vivo. **A** Western blot and **B** qPCR analysis results validated the regulation of CMTM6 in HuH-7 and HepG2. **C** CCK-8 assay and **D** colony-formation assay showing the cell growth of the indicated HCC cells. **E** Evaluation of the effects of CMTM6 on HCC cells proliferation by BrdU incorporation analysis. **F** Effects of CMTM6 on cell spontaneous apoptosis as assessed by flow cytometric analysis. **G** Images of the dissected tumors from the nude mice. **H** The tumor weight of each group is shown. **I** The tumor growth curves of each group are shown. All in vitro experiments were performed in triplicate. The error bars represent the mean ± SD. *P < 0.05; **P < 0.01; ***P < 0.001; ****P < 0.0001; ns means non-significant.

Since CMTM6 expression was correlated with clinical prognosis in HCC patients, we investigated the effect of CMTM6 on the growth of HCC cells. We established stable CMTM6-overexpressing cell lines using HuH-7 and SK-Hep-1 cells and knocked down CMTM6 in HepG2 and Hep3B cells (Fig. 2A, B and S2A, B). The CCK-8 assays revealed that CMTM6 markedly suppressed the growth of the indicated cells (Fig. 2C and S2C). Likewise, colony formation assays demonstrated that the foci formation frequency of cells was decreased with the overexpression of CMTM6, and increased with the knockdown of CMTM6 (Fig. 2D and S2D). It has been reported that suppression of cancer cell growth could be caused by inhibition of cell proliferation, induction of apoptosis, or both [23]. To determine whether the difference in cell viability was due to a difference in proliferation, we performed BrdU incorporation assays and found that CMTM6 markedly suppressed cell proliferation (Figs. 2E and S2E). In contrast, the regulation of CMTM6 exhibited slight effect on spontaneous apoptosis of HCC cells (Figs. 2F and S2F, G). Therefore, we concluded that CMTM6 suppressed HCC cell growth mainly by inhibiting proliferation. Besides, CMTM6 was recently reported as stemness regulator in head and neck squamous cell carcinoma [9]. However, in our study, we found that CMTM6 had no significant influence on the stemness of HCC (Fig. S3). Then we evaluated the effects of CMTM6 on HCC tumor growth in vivo by xenograft model. As shown in Fig. 2G, H, mice injected with HuH7/OE-CMTM6 cells developed significantly smaller tumors than those injected with HuH7/OE-NC cells. Likewise, larger tumors were observed in the CMTM6-depleted groups. The tumor growth curves of NCG mice in each group further confirmed these results (Fig. 2I). Moreover, IHC analysis revealed that the expression of Ki67 were remarkably reduced in HuH-7/OE-CMTM6 tumors and increased in HepG2/shCMTM6 tumors compared to their respective control groups (Fig. S4). These results provide strong evidence that CMTM6 plays a critical role in the suppression of tumor growth of HCC both in vitro and in vivo.

### CMTM6 induces G0/G1 cell cycle arrest by interacting with p21 and regulating its protein expression levels

**Fig. 3:**
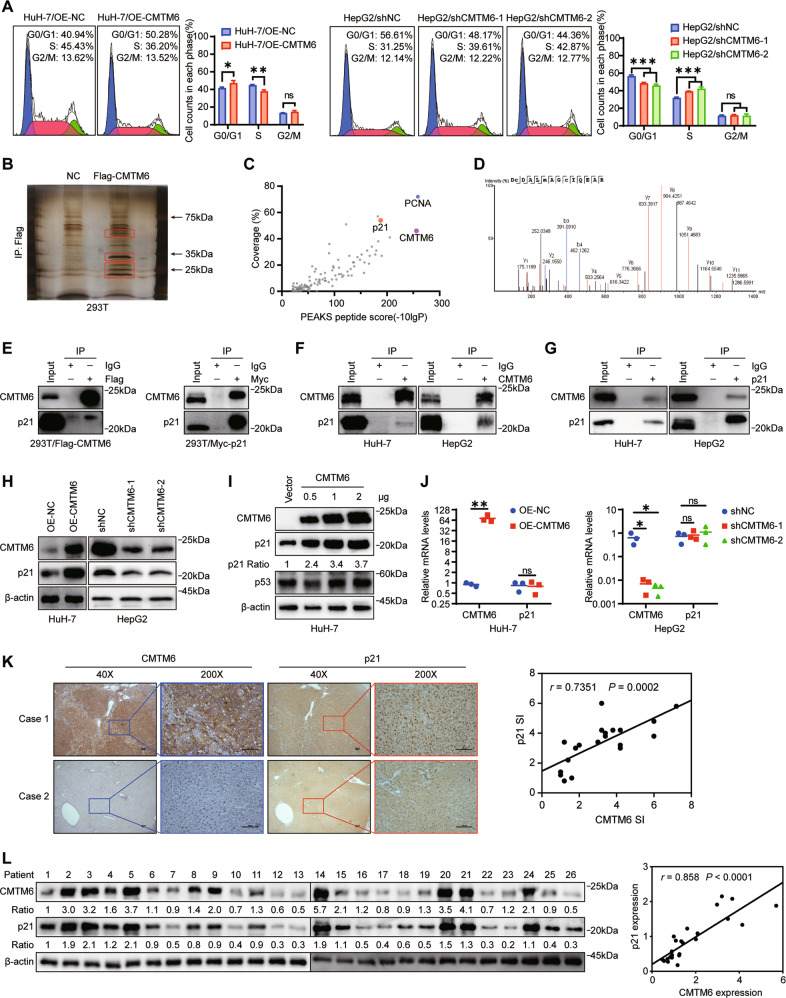
CMTM6 induces G0/G1 cell cycle arrest by interacting with p21 and regulating its protein levels. **A** Cell cycle distribution of the indicated HCC cells as assessed by flow cytometric analysis. **B** 293 T cells transfected with either empty vectors or vectors expressing Flag-CMTM6 were subjected to immunoprecipitation with an anti-Flag antibody, followed by silver staining. The red boxes indicated the overtly differential bands send to LC-MS/MS analysis. **C** Scatterplots display proteins identified by LC-MS/MS. **D** The best unique peptide-spectrum matches (PSM) of p21. **E** 293 T cells transfected with plasmids expressing either Flag-CMTM6 or Myc-p21 were subjected to immunoprecipitation with control IgG, anti-Flag (left) or anti-Myc (right) antibody. **F** Immunoprecipitation of HuH-7 and HepG2 cell lysates with control IgG or anti-CMTM6 antibody. **G** Immunoprecipitation of HuH-7 and HepG2 cell lysates with control IgG or anti-p21 antibody. **H** Western blot analysis of CMTM6 and p21 expression in the indicated cells. **I** Increasing amounts of CMTM6 plasmids were transfected into HuH-7 cells for 72 h. Protein levels of CMTM6 and p21 were then evaluated by Western blot analysis. **J** qPCR analysis of CMTM6 and p21 expression in the indicated cells. **K** Representative IHC staining images of p21 in tumor samples with high expression and low expression of CMTM6 (left). Statistical analysis of the correlation between p21 and CMTM6 expression levels detected by IHC staining (right). **L** Western blot analysis of p21 and CMTM6 protein expression in HCC tumor samples(left). Statistical analysis of the correlation between p21 and CMTM6 expression levels detected by Western blotting (right). *P < 0.05; **P < 0.01; ***P < 0.001; ns means non-significant.

To elucidate the potential mechanisms by which CMTM6 inhibits HCC cell proliferation, we evaluated the possible effects of CMTM6 on the cell cycle distribution. As shown in Figs. 3A and S5A, CMTM6 significantly induced HCC cells arrest in the G0/G1 phase, while knockdown of CMTM6 significantly promoted the G1/S phase transition. In order to identify cell cycle related proteins that interact with CMTM6, we performed silver staining after immunoprecipitation assays and analyzed the overtly differential bands by liquid chromatography-tandem mass spectrometry (LC-MS/MS, Fig. 3B). As shown in Fig. 3C, D, p21 is identified as one of the highest scoring proteins. To confirm the LC-MS/MS results, Flag-CMTM6 or Myc-p21 plasmids were transfected into 293 T cells, and CoIP assays were performed using an anti-Flag or anti-Myc antibody. As shown in Fig. 3E, p21 was detected in the Flag-CMTM6 immunoprecipitates and CMTM6 was detected in the Myc-p21 immunoprecipitates. Although PCNA was also detected in the CMTM6-bound protein complexes by LC-MS/MS, repearted CoIP assays followed by Western blot analysis only found PCNA in the Myc-p21 immunoprecipitates but not in the Flag-CMTM6 immunoprecipitates, implying that PCNA might indirectly bind to CMTM6 via p21 (Fig. S5 B, C). In addition, we also confirmed the association between endogenous CMTM6 and p21 in HuH-7 and HepG2 cells by CoIP with anti-CMTM6 or anti-p21 antibody (Fig. 3F, G).

Protein–protein interactions are known to play key roles in regulating p21 expression levels, thus we investigated whether CMTM6 influences the expression of p21. Western blot analysis showed that CMTM6 significantly increased the protein expression of p21, while the expression levels of p21 were markedly downregulated with the depletion of CMTM6 (Fig. 3H). Furthermore, increasing CMTM6 expression induced the upregulation of p21 in a dose-dependent manner regardless of the p53 status (Fig. 3I). On the contrary, neither overexpression nor knockdown of CMTM6 altered the mRNA level of p21, indicating that CMTM6 did not affect the transcription of p21 (Fig. 3J and Fig. S5D). Together, these results imply that CMTM6 directly binds to p21 and positively regulates its protein expression by post-transcriptional modification. Besides, IHC and Western blot analysis revealed a positive correlation between CMTM6 and p21 expression in human HCC samples (*r* = 0.7351, *P* = 0.0002, Figs. 3K, L and S5E), whereas statistical analysis of the qPCR assays and datasets from TCGA database showed no significant correlation between CMTM6 and p21 mRNA expression. (Fig. S5F, G).

**Fig. 4:**
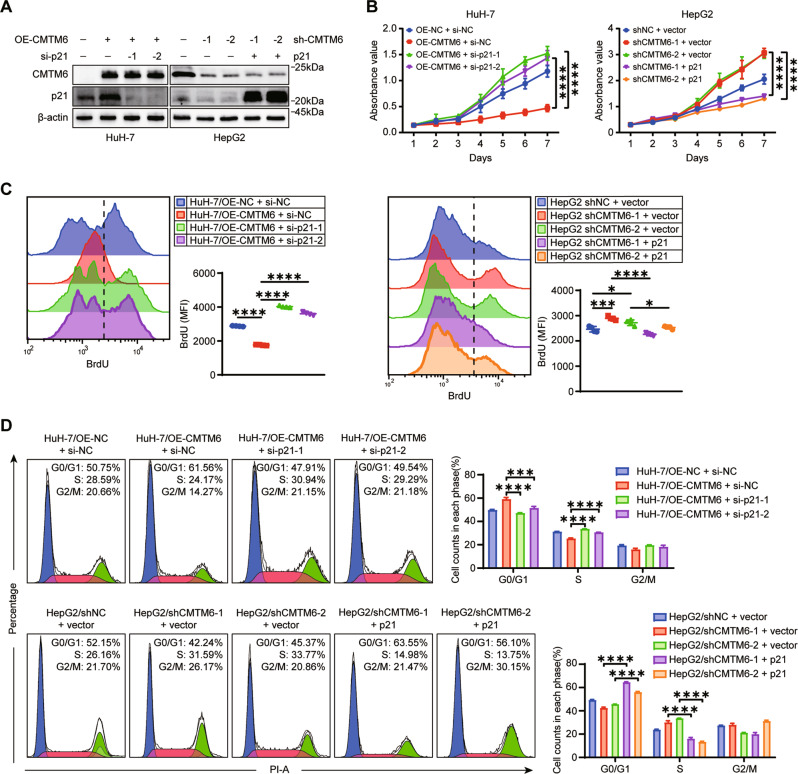
p21 was responsible for the CMTM6-mediated tumor suppression. **A** Modulation of the CMTM6 and p21 expression in HCC cells was validated by Western blot analysis. **B** CCK-8 assays and (**C**) BrdU corporation assay showing the cell growth of the indicated HCC cells. **D** Cell cycle distribution of the indicated HCC cells as assessed by flow cytometric analysis. The experiments were performed in triplicate. The error bars represent the mean ± SD. *P < 0.05; ***P < 0.001; ****P < 0.0001.

p21 is widely recognized as a key negative regulator of cell cycle progression. In this study, we also confirmed that p21 could suppress the proliferation of HCC cell (Fig. S6). To further determine whether the levels of p21 were responsible for the CMTM6-mediated effects, we silenced p21 expression in CMTM6-overexpressing HuH-7 cells and ectopically overexpressed p21 in CMTM6-depleted HepG2 cells (Fig. 4A). As shown in Fig. 4B, C, the results of the CCK-8 assays and BrdU incorporation assays revealed that overexpression of CMTM6 significantly inhibited cell viability and proliferation of HuH-7 cells, which could be rescued by p21 knockdown. Similarly, cell viability and proliferation of HepG2 cells were prominently accelerated by CMTM6 depletion, and these effects were abolished by extra p21 expression. In addition, inversely regulation of p21 in HCC cells could block the effects of CMTM6 on cell cycle distribution (Fig. 4D). Collectively, these data indicated that CMTM6 suppresses HCC cell growth and blocks the G1/S phase transition by regulating the levels of p21.

### CMTM6 protects p21 from ubiquitin-mediated degradation in a cell-cycle–independent manner

**Fig. 5:**
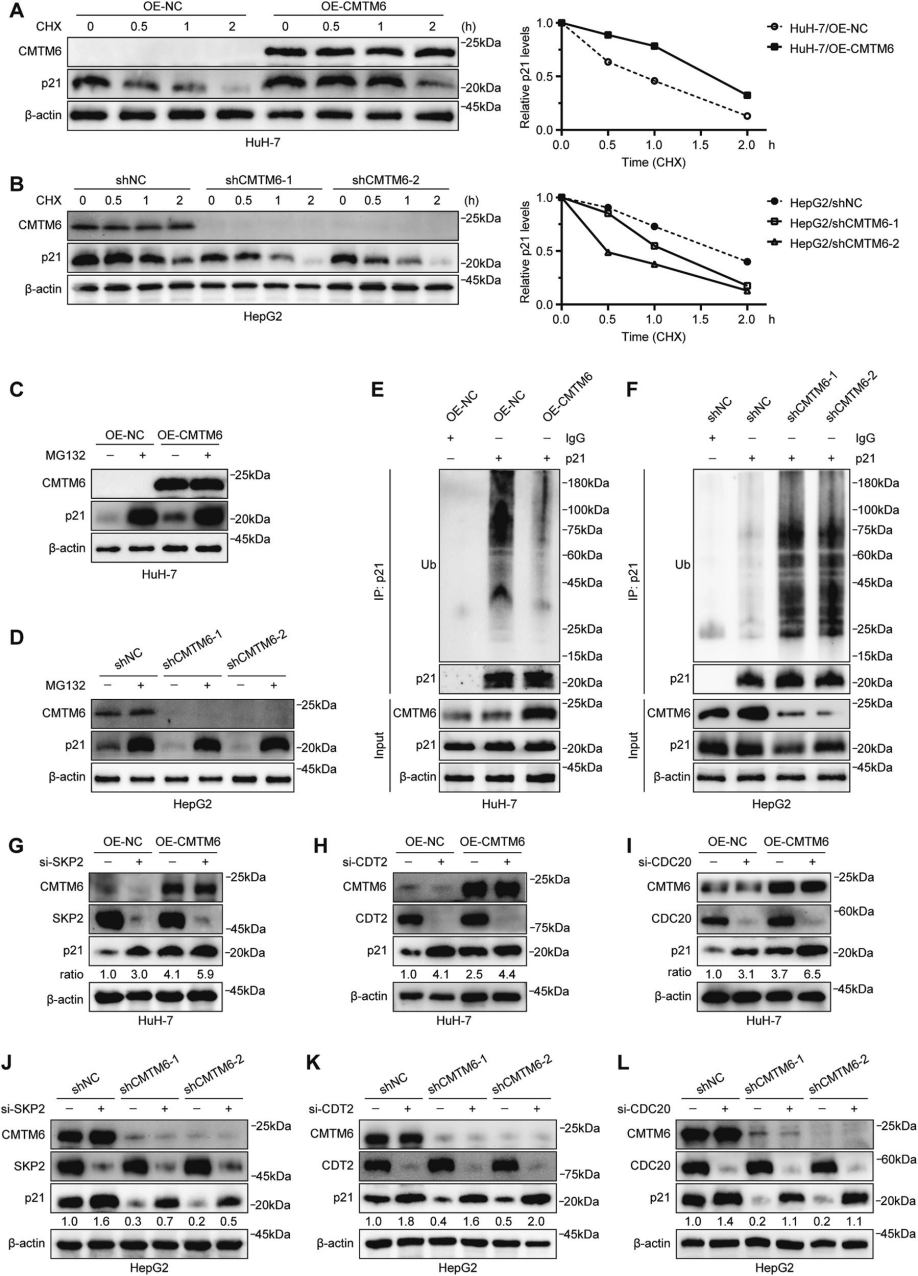
CMTM6 protects p21 from ubiquitin-mediated degradation throughout the cell cycle. **A** HuH-7 and (**B**) HepG2 cells transfected with the indicated constructs were treated with 10 μM CHX, collected at the specific time points and immunoblotted with anti-CMTM6, anti-p21, and anti-β-actin antibodies (left). Quantification of the p21 levels relative to β-actin expression is shown (right). **C** HuH-7 and (**D**) HepG2 cells transfected with the indicated constructs were treated with DMSO or MG132 (20 μM) for 6 h, and the expression of CMTM6, p21 and β-actin was analyzed by Western blotting. **E** HuH-7 and **F** HepG2 cells transfected with the indicated constructs were treated with MG132 (20 μM) for 6 h and then subjected to immunoprecipitation with control IgG or anti-p21 antibody. The total cell lysates were probed with anti-CMTM6, anti-p21 and anti-β-actin antibodies, while the immunoprecipitates were probed with anti-Ubiquitin and anti-p21 antibodies. **G**–**I** HuH-7 cells infected with the indicated constructs were transfected with scrambled, SKP2 (**G**), CDT2 (**H**), or CDC20 (**I**) siRNA for 48 h, and then the cell lysates were prepared and analyzed by Western blotting. **J**–**L** HepG2 cells infected with indicated shRNAs were transfected with scrambled, SKP2 (**J**), CDT2 (**K**), or CDC20 (**L**) siRNA for 48 h, and then the cell lysates were prepared and analyzed by Western blotting.

Given that p21 is an unstable protein that can be rapidly degraded by the ubiquitin-proteasome pathway [21], we wondered whether CMTM6 could improve the stability of p21. To this end, we treated the indicated cells with 10 μM cycloheximide (CHX) to block the protein biosynthesis, and collected the cells at the indicated time points to analyze the degradation levels of p21. Western blot analysis showed that overexpression of CMTM6 considerably extended the half-life of the p21 protein (Fig. 5A), while knockdown of CMTM6 significantly shortened its half-life (Fig. 5B). Besides, the change of p21 protein levels induced by CMTM6 modulation could be blocked by the proteasome inhibitor MG132 (Fig. 5C, D). Thus, we suggest that CMTM6 stabilizes p21 by repressing its ubiquitin-proteasome proteolysis. To this end, we treated the indicated cells with MG132 (20 μM) for 6 h and performed immunoprecipitation analysis with an anti-p21 antibody. The results showed that overexpression of CMTM6 significantly reduced the polyubiquitination levels of p21 (Fig. 5E), whereas knockdown of CMTM6 markedly increased the amount of ubiquitinated p21 (Fig. 5F). These results indicated that CMTM6 reduced the polyubiquitination levels of p21 and suppressed its degradation in HCC cells.

It is widely recognized that p21 has a cell cycle arrest effect throughout the cell cycle process [24], thus we investigated when and how CMTM6 influences the ubiquitination of p21. The indicated cells were synchronized at each cell cycle phase (Fig. S7A) and the expression of p21 was measured by Western blotting. The results showed that CMTM6 positively regulated the expression of p21 in all phases of the cell cycle (Fig. S7B). Three E3 ubiquitin ligase complexes, namely SCF^SKP2^, CRL4^CDT2^, and APC/C^CDC20^, have been reported to induce p21 ubiquitination and proteolysis in specific phases of the cell cycle [25–27]. Therefore, we also examined whether the effect of CMTM6 on p21 was related to SCF^SKP2^, CRL4^CDT2^, and APC/C^CDC20^. Regulation of CMTM6 levels had no effect on the expression of SKP2, CDT2 and CDC20, and vice versa (Fig. S7C–F). Strikingly, knocking down SKP2, CDT2 or CDC20 had a slight influence on the levels of p21 in HuH-7/OE-CMTM6 cells, but significantly increases it in the control cells (Fig. 5G–I). Likewise, silencing SKP2, CDT2 or CDC20 in HepG2 cells resulted in a much stronger accumulation of p21 in CMTM6-deficient cells than in CMTM6-proficient cells (Fig. 5J–L). Furthermore, HuH-7 and HepG2 cells stably overexpressing p21 were transfected with CMTM6 plasmids or CMTM6 small interfering RNAs, respectively. CoIP assays showed that CMTM6 decreased the binding of SKP2, CDT2 and CDC20 with p21 (Fig. S7G), which suggested that CMTM6 inhibited p21 ubiquitination via competitively binding with p21 against SCF^SKP2^, CRL4^CDT2^ and APC/C^CDC20^. Overall, these results indicated that CMTM6 functioned as a molecular partner of p21 and protected it from ubiquitination mediated by SCF^SKP2^, CRL4^CDT2^ or APC/C^CDC20^ in a cell-cycle-independent manner.

### CMTM6 reduces the phosphorylation of RB and downregulates the transcriptional activity of E2F1

**Fig. 6:**
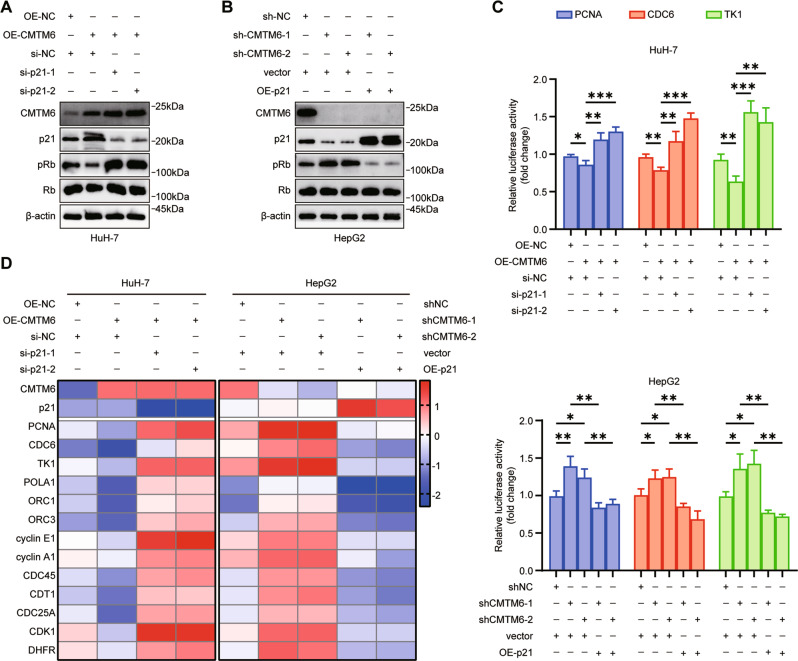
CMTM6 reduced the phosphorylation of RB and downregulates E2F1 transcription factors activity. **A** The HuH-7 cells stably overexpressing CMTM6 were transfected with scramble or two independent p21 siRNAs. The expression of p21, pRB and total RB was detected by Western blot analysis. **B** The HepG2 cells with stable CMTM6 depletion were transfected with empty vectors or vectors expressing p21. The expression of p21, pRB and total RB was detected by Western blot analysis. **C** Dual-luciferase reporter assays revealing the transcriptional activity of E2F1 on the promoters of CDC6, PCNA and TK1 in the indicated cells. **D** qPCR analysis of the mRNA levels of the candidate downstream targets of E2F1 in the indicated cells. The error bars represent the mean ± SD. *P < 0.05; **P < 0.01; ***P < 0.001.

Given that the ability of p21 to block the G1/S phase transition primarily depends on its inhibitory effects on various CDK-cyclin complexes, which is essential for the phosphorylation of RB [28, 29], we hypothesized that CMTM6 inhibits the G1/S phase transition mainly by suppressing the pRB/E2F pathway. Western blot analysis showed that overexpression of CMTM6 in HuH-7 cells significantly reduced the expression of pRB protein (Fig. 6A), which could be rescued by silencing of p21 (Fig. 6A). Similarly, the upregulation of pRB was induced by knocking down CMTM6 and reversed by ectopic overexpression of p21 (Fig. 6B). Hypophosphorylated RB binds to E2F transcription factors and blocks the transcriptional activation of the genes required for the transition from the G1 to S phase. Thus, we took E2F1 as the representative to examine the effect of CMTM6 on the activity of E2F transcription factors. Dual-luciferase reporter assays indicated that overexpression of CMTM6 significantly reduced the transcriptional activity of E2F1 on the promoters of CDC6, PCNA and TK1, while knockdown of CMTM6 upregulated its transcriptional activity. Similar to these results, reverse regulation of p21 expression was found to block the effect of CMTM6 on the E2F1 transcription factor (Fig. 6C). In addition, qPCR analysis revealed that overexpression and knockdown of CMTM6 negatively regulated the mRNA expression of the representative E2F target genes involved in DNA synthesis and S phase entry. Likewise, these CMTM6-mediated mRNA level changes could be reversed by the inverse regulation of p21 (Fig. 6D). Overall, these findings suggested that CMTM6 induces the dephosphorylation of RB protein and inhibits the transcriptional activity of E2F transcription factors by acting on p21.

### CMTM6 sensitizes HCC to Dox and DDP and its downregulation predicts poor prognosis of TACE outcome

**Fig. 7:**
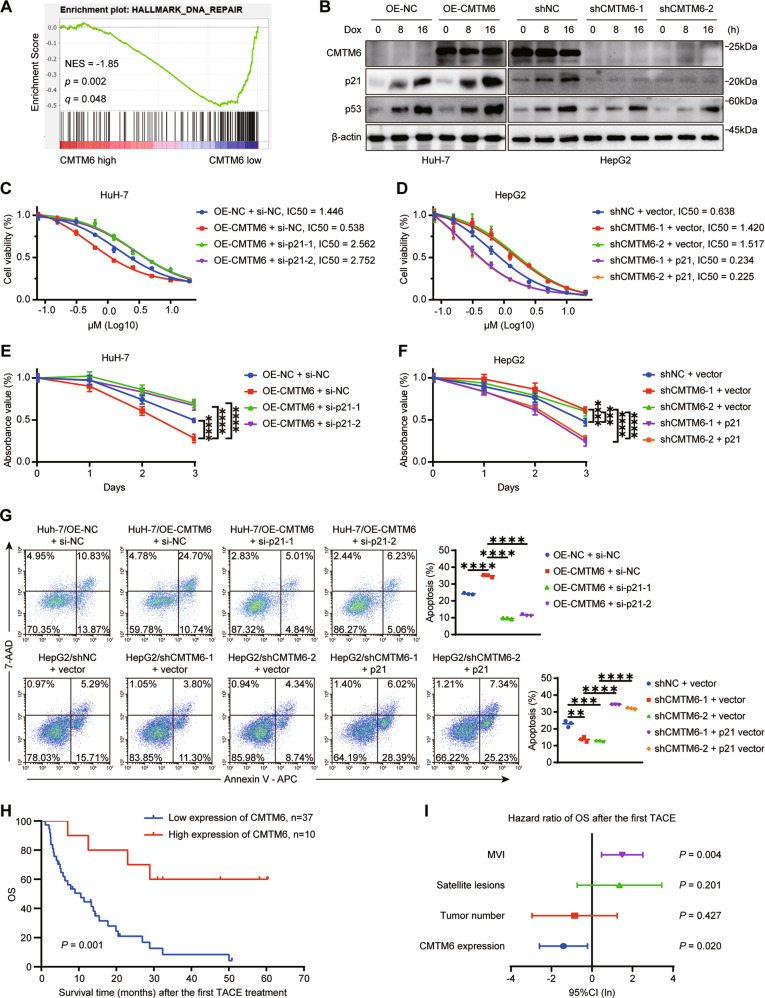
CMTM6 sensitizes HCC to Dox and its downregulation predicts poor prognosis of TACE treatment outcome. **A** GSEA plot showing that CMTM6 expression is negatively correlated with DNA repair in HCC. **B** The indicated cells were treated with 0.2 μM Dox for 0, 8 or 16 h. Cell lysates were then extracted and subjected to Western blot analysis. **C** HuH-7 and **D** HepG2 cells transfected with the indicated constructs were treated with Dox at different concentrations for 48 h and cell viability was then measured by the CCK-8 assay. **E** HuH-7 and **F** HepG2 cells transfected with the indicated constructs were treated with 0.2 μM Dox for different time periods and cell viability was measured by the CCK-8 assay. **G** HuH-7 (top) and HepG2 (bottom) cells transfected with the indicated constructs were treated with 0.5 μM Dox for 48 h and cell apoptosis was assessed by flow cytometric analysis. **H** Kaplan–Meier analysis of OS after the first TACE treatment according to CMTM6 expression status. **I** Multivariate analysis of CMTM6 expression and clinical variables in HCC patients who received TACE treatment. The experiments were performed in triplicate. The error bars represent the mean ± SD. **P < 0.01; ***P < 0.001; ****P < 0.0001.

p21 came into the spotlight as an inhibitor of cell cycle and a modulator of DNA repair. The effect of CMTM6 on p21 regarding the former function has been examined above, we next explored whether CMTM6 could influence the ability of p21 to regulate DNA repair. The gene set enrichment analysis (GSEA) plot revealed that the levels of CMTM6 were negatively related to DNA repair (*P* = 0.002, Fig. 7A). To determine whether CMTM6 is involved in the DNA damage-mediated regulation of p21, we treated the indicated cells with 0.2 μM Dox or 10 μM DDP, which are chemotherapeutic agents commonly used in TACE treatment for HCC, for 0, 8 and 16 h, separately. As shown in Figs. 7B and S8A, Dox or DDP treatment led to upregulation of p21 levels in HCC cells, which could be enhanced by overexpression of CMTM6 and abolished by CMTM6 depletion. Notably, the expression of CMTM6 remained consistent in the presence or absence of genotoxic treatment. These results indicated that CMTM6 could stabilize p21 in response to DNA damage. To determine the influence of CMTM6 on Dox and DDP sensitivity, cells were exposed to the indicated concentrations of Dox or DDP for 48 h. Cell viability assays showed that CMTM6 promoted the sensitivity of HCC cells to Dox and DDP and decreased their half maximal inhibitory concentration (IC_50_) value, whereas inverse regulation of p21 expression could reverse the effect of CMTM6 on Dox and DDP sensitivity. (Figs. 7C, D, S8B, C and S9A, B). Additional results confirmed that CMTM6 inhibited the viability of HCC cells under exposure to Dox (Figs. 7E, F and S9C, D) or DDP (Figs. S8D, E and S9F, G) treatments in a time-dependent manner. Moreover, we also investigated the effect of CMTM6 on apoptosis induced by DNA-damaging agents. Compared with the control cells, CMTM6-proficient cells exhibited a significant increase in the apoptosis rate after 48 h exposure to Dox or DDP. In contrast, ablation of p21 fully reversed the apoptosis-promoting effect of CMTM6 overexpression. Likewise, knockdown of CMTM6 markedly reduced the rate of apoptotic cells, which could be rescued by restoring p21 expression (Figs. 7G, S8F and S9E, H). These findings suggested that CMTM6 sensitizes HCC cells to Dox and DDP by promoting p21 accumulation.

According to the BCLC staging system, TACE is the first-line of treatment for patients with intermediate stage HCC [30]. Genotoxic drugs (e.g., Dox or DDP) emulsified in the oily radio-opaque agent lipiodol are recommended to be applied in TACE [4]. Given that CMTM6 could improve the sensitivity of HCC to Dox and DDP, we wondered whether CMTM6 was associated with the prognosis of the TACE treatment. To investigate this potential association, we explored the levels of CMTM6 in 47 primary tumor specimens obtained from patients who underwent surgery and received TACE treatment after postoperative recurrence. Noteworthy, patients with high CMTM6 expression had significantly longer OS (Fig. 7H). Moreover, multivariate analysis revealed that CMTM6 expression status was an independent indicator of OS after the first TACE treatment (Fig. 7I and Table S6). Collectively, the above results indicated that CMTM6 sensitizes HCC cells to Dox and DDP and might be a potential predictor of TACE treatment outcome.

## Discussion

CMTM6 is a ubiquitously expressed protein that belongs to a family of eight MARVEL domain-containing proteins [6]. Most studies on CMTM6 have focused on its role in the regulation of the immune response [7, 8]. Recently, CMTM6 has been found to play multiple roles in tumor progression but different studies have reached completely opposite conclusions. CMTM6 may serve as either a promoter or a suppressor in different tumors [9–15]. In this study, we demonstrated that the expression of CMTM6 was significantly downregulated in HCC and that patients with low CMTM6 expression had a higher risk of death and recurrence, which supports the results previously reported by Zhu et al. [31]. Additional studies, for the first time, indicated that CMTM6 induced cell cycle arrest and repressed the proliferation of HCC cells both in vitro and in vivo. The above data strongly suggest that CMTM6 functions as a tumor suppressor in HCC. However, Huang et al. showed that CMTM6 promoted migration, invasion and EMT in HCC and poor prognosis of HCC was associated significantly with higher CMTM6 expression [32]. Yugawa et al. also found that CMTM6 stabilizes PD-L1 expression and is a prognostic impact factor in HCC [33]. We speculated that the contradictory results are mainly due to the different clinicopathological features of the patients. Huang et al. focused on the migration and invasion of HCC and enrolled the majority of the patients with microvascular invasion. The majority of HCC in China occurs in patients with hepatitis B virus infection, while Yugawa et al. enrolled the patients from Japan and most of the patients were HBs-Ag negative. On the contrary, we mainly explore the growth of HCC and most of the patients in our study had no microvascular invasion and portal vein tumor thrombus, but their HBs-Ag was positive.

The cyclin-dependent kinase inhibitor p21 (also named p21^WAF1/Cip1^ or *CDKN1A*) is well established as a key negative regulator of cell cycle progression [18]. In this study, we presented the first evidence that p21 directly interacts with CMTM6. Modulation of CMTM6 positively regulated p21 protein expression by post-transcriptional modification. IHC and Western blot analysis of the HCC samples also demonstrated the positive association between the protein levels of CMTM6 and p21. Moreover, inversely regulation of p21 could block the suppressing effects of CMTM6 on HCC cells, confirming that CMTM6 functioned as a tumor suppressor mainly by acting on p21. Under normal growth conditions, p21 is an unstable protein [24] and its stability is regulated mainly by post-translational modifications, such as phosphorylation and ubiquitination [21, 34]. Regarding the ubiquitin-dependent pathway, three E3 ubiquitin ligase complexes, namely SCF^SKP2^, CRL4^CDT2^ and APC/C^CDC20^, account for the ubiquitination of p21 in the G1/ S transition, S phase and G2/M transition [25–27, 35], respectively. In this study, we found that CMTM6 significantly prolonged the half-life of p21 and the change of p21 expression induced by CMTM6 regulation could be abolished by the proteasome inhibitor MG132. Ubiquitination assays revealed that CMTM6 reduced the poly ubiquitination levels of p21. In addition, silencing SKP2, CDT2 or CDC20 resulted in a stronger increase in p21 levels in CMTM6-deficient than in CMTM6-proficient cells. These experiments together revealed the interesting findings that CMTM6 interacts with p21 and protects it from ubiquitin-dependent proteolysis carried out by SCF^SKP2^, CRL4^CDT2^ and APC/C^CDC20^.

It is well known that p21 inhibits G1/S phase transition primarily by physically binding to cyclin-CDK4/6 and -CDK2 and inhibiting their phosphorylation activity on RB [36, 37]. Hypophosphorylated forms of RB bind to E2F transcription factors and restrict their transcriptional activity [38]. Consistent with previous study, our study indicated that by maintaining the protein levels of p21, CMTM6 could reduce the phosphorylation of RB, reduced the activity of E2F1 and inhibited the transcription of a set of typical G1/S cell cycle transition genes. Together, these results demonstrated that the downregulation of CMTM6 in HCC cells activates the pRB/E2F1 pathway by decreasing the levels of p21, which consequently promotes G1/S phase transition in HCC cells.

Another well-known function of p21 is to regulate DNA repair. Studies on the effects of p21 on DNA repair have reached contradictory conclusions that it plays a role as either an enhancer or a suppressor of various DNA repair pathways [24, 39, 40]. In this study, GSEA of the TCGA datasets showed a negative association between CMTM6 and DNA repair. We found that CMTM6 significantly enhanced the upregulation of p21 induced by Dox and DDP. By maintaining p21 expression, CMTM6 promoted the sensitivity of HCC cells to Dox and DDP. Although the precise mechanism by which CMTM6 enhances the efficacy of Dox and DDP needs to be further investigated, we believe that CMTM6 sensitizes HCC cells to Dox and DDP at least in part by its ability to stabilize p21 and consequently impair DNA repair.

TACE is recommended as the standard care for intermediate stage HCC (BCLC B) [4] and an effective treatment for patients who experience postoperative recurrence. Despite considerable advances in TACE techniques, the survival outcome of patients managed with this technique is variable [41]. Patients with postoperative recurrence often do not have the option of curative surgery, their recurrent tumor specimens are usually unavailable. However, it has been reported that the majority of recurrent HCCs derive from the clonal lineage of the initial tumor [42]. It is meaningful to find biomarkers from the initial tumors that can predict the therapeutic efficacy of TACE for recurrent HCC. In this study, we found that lower levels of CMTM6 in the initial tumors were correlated with poor prognosis after TACE. Since genotoxic agent treatment can induce p21 upregulation but has no influence on the levels CMTM6, we speculate that the expression levels of CMTM6 but not p21 in the initial tumors might be a potential biomarker to reflect the sensitivity of tumor to TACE. Our study constructively provides a new clinical strategy for HCC patients with postoperative recurrence. Even if the recurrent tumor specimens are unavailable, the expression level of CMTM6 in the initial tumors can be used to preliminary determine whether a patient is suitable for TACE treatment. Patients with high CMTM6 expression are more likely to benefit from TACE treatment.

There are some limitations in our study. First, we focused on the downstream pathway of CMTM6 without referring to the regulation of CMTM6 in HCC. The reason for the down-regulation of CMTM6 in HCC needs to be further explored. Besides, we speculate that whether the patient is infected with HBV is the reason why our results contradict those of Yugawa et al. However, we have not verified our speculation nor explored whether HBV could regulate the expression or function of CMTM6. Despite these limitations, our study not only contribute to our understanding of the role of CMTM6 in tumor growth in HCC, but will also lead to the characterization of a specific HCC patients’ group with CMTM6 overexpression who may benefit from TACE.

## Materials and methods

### Patients and specimens

A total of 167 paraffin-embedded HCC samples were obtained from HCC patients who underwent hepatectomy at Sun Yat-sen University Cancer Center (Guangzhou, China) from November 2005 to November 2011. The diagnosis was histologically confirmed by pathological examination, and none of the patients had received any antitumor therapy before surgery. In addition, 34 fresh specimens of HCC tissues paired with non-tumorous liver tissues were collected from HCC patients who underwent hepatectomy between December 2017 and May 2018. Written informed consent was obtained from all patients before sample collection, and ethics approval for the use of the specimens was provided by the Internal Review and the Ethics Boards of the Sun Yat-Sen University Cancer Center.

### Western blot and immunoprecipitation analysis

Total proteins were isolated from HCC tissues and cells using RIPA lysis buffer (MilliporeSigma, Burlington, MA, USA) with freshly added protease inhibitor cocktail (Roche AG, Basel, Switzerland). The protein concentration was determined using the Detergent Compatible Bradford Protein Assay Kit (Beyotime, shanghai, China). 10 μg of the protein extracts was separated on 10% gels by standard sodium dodecyl sulfate/polyacrylamide gel electrophoresis (SDS/PAGE) and transferred to polyvinylidene fluoride (PVDF) membranes (MilliporeSigma). The membranes were sealed in 5% nonfat milk, probed by primary antibodies overnight, and then incubated with secondary antibody for 1 h. An enhanced chemiluminescence (ECL) reagent (Pierce, Rockford, IL, USA) was used to visualize the immunoblots. The primary antibodies used in this experiment are listed in Table S2.

Cell lysates for (co)immunoprecipitation experiments were prepared by lysing cells in immunoprecipitation (IP) Lysis Buffer (Thermo Fisher Scientific Inc.) containing protease inhibitor cocktail (Roche AG). Then, 1 mg of the clarified lysates was incubated overnight at 4 °C with 2 μg of either the appropriate primary antibodies or an isotype-matched negative control IgG. Subsequently, the sample-antibody mixtures were rotated together with Protein A/G Magnetic Beads (MedChemExpress (MCE) LLC., Monmouth Junction, NJ, USA) for 2 h at 4 °C and then washed five times with IP Lysis Buffer and collected by magnetic separation. The samples were ultimately eluted with Lane Marker Reducing Sample (Invitrogen, Carlsbad, CA, USA) and further assessed by Western blot analysis.

### Protein half-life assay

Cells were treated with 10 μM cycloheximide (CHX; MCE) for various periods of time to block protein synthesis. Crude protein extracts were prepared, and the protein levels were determined by Western blot analysis.

### Ubiquitination assay

The indicated cells were treated with 20 μM MG132 (MilliporeSigma) for 6 h before harvesting. Subsequently, cells were washed with PBS and lysed with IP lysis buffer containing protease inhibitor cocktail (Roche AG) and 10 μM N-ethylmaleimide (MCE) on ice for 30 min. The lysates were heated at 95 °C for 10 min and then quenched by incubating with nine volumes of quenching buffer (0.5% Triton X-100, 20 mM Tris-HCl (pH 8.0), 137 mM NaCl, 10% glycerol, 2 mM EDTA) at 4 °C for 30 min with rotation. Afterwards, the lysates were centrifuged at 12,000 rpm for 15 min at 4 °C, and then incubated with the anti-p21 antibody overnight. Subsequently, the samples were rotated together with Protein A/G Magnetic Beads (MCE) for an additional 1 h at 4 °C. Then, the beads were washed three times and the bound proteins were released from the beads by boiling the beads in SDS/PAGE sample buffer, and analyzed by immunoblotting with anti-Ubiquitin mAb.

### Cell cycle synchronization

To synchronize HuH-7 and HepG2 cells at the G1/S phase border, cells were treated with 2 mM thymidine (MCE) for 18 h. After washing twice with PBS, cells were cultured in complete growth medium for 9 h, followed by addition of 2 mM thymidine for another 16 h. After rinsing twice with PBS, cells were collected immediately (G1/S phase border) or cultured in complete growth medium for 3 h (S-phase cells), 6 h (G2-phase cells), or treated with 100 ng/mL of nocodazole for 11 h (M-phase cells). Cells were collected and analyzed by flow cytometry and Western blotting.

### Statistical analysis

Data were expressed as the mean ± standard deviation (SD). Survival analysis was conducted using the Kaplan–Meier method. The χ2 test was performed to analyze the relationship between CMTM6 expression and the clinicopathological characteristics. Based on the variables selected by univariate analysis, the multivariate Cox proportional hazards model was used to determine the independent prognostic factors of HCC. The association between CMTM6 and p21, or Ki67 expression in HCC tissues was calculated using the Pearson correlation test. The differences between the groups were evaluated using the Student two-tailed *t* test and one-way analysis of variance (ANOVA). *P* < 0.05 was considered statistically significant. The statistical analysis and figure generation were performed using the Prism 8.0 software (GraphPad Software, Inc., San Diego, CA, USA) and SPSS version 23.0 (IBM Corporation, Armonk, NY, USA).

Other materials and methods of this study were listed in the Supplementary Materials and Methods.

## Supplementary information

Supplementary materials and methods

Supplemental Tables

Supplemental figure legends

Figure S1

Figure S2

Figure S3

Figure S4

Figure S5

Figure S6

Figure S7

Figure S8

Figure S9

uncropped western blots

Reproducibility checklist

## Data Availability

The data that support the findings of this study are available from the corresponding author upon reasonable request.

## References

[1] Siegel RL, Miller KD, Fuchs HE, Jemal A (2021). Cancer Statistics, 2021. CA Cancer J Clin.

[2] Bruix J, Reig M, Sherman M (2016). Evidence-based diagnosis, staging, and treatment of patients with hepatocellular carcinoma. Gastroenterology.

[3] Gerbes A, Zoulim F, Tilg H (2018). Gut roundtable meeting paper: selected recent advances in hepatocellular carcinoma. Gut.

[4] Sieghart W, Hucke F, Peck-Radosavljevic M (2015). Transarterial chemoembolization: modalities, indication, and patient selection. J Hepatol.

[5] Crissien AM, Frenette C (2014). Current management of hepatocellular carcinoma. Gastroenterol Hepatol (N. Y).

[6] Han W, Ding P, Xu M, Wang L, Rui M, Shi S (2003). Identification of eight genes encoding chemokine-like factor superfamily members 1–8 (CKLFSF1-8) by in silico cloning and experimental validation. Genomics.

[7] Mezzadra R, Sun C, Jae LT, Gomez-Eerland R, de Vries E, Wu W (2017). Identification of CMTM6 and CMTM4 as PD-L1 protein regulators. Nature.

[8] Burr ML, Sparbier CE, Chan YC, Williamson JC, Woods K, Beavis PA (2017). CMTM6 maintains the expression of PD-L1 and regulates anti-tumour immunity. Nature.

[9] Chen L, Yang QC, Li YC, Yang LL, Liu JF, Li H (2020). Targeting CMTM6 suppresses stem cell-like properties and enhances antitumor immunity in head and neck squamous cell carcinoma. Cancer Immunol Res.

[10] Zheng Y, Wang C, Song A, Jiang F, Zhou J, Li G (2020). CMTM6 promotes cell proliferation and invasion in oral squamous cell carcinoma by interacting with NRP1. Am J Cancer Res.

[11] Li X, Chen L, Gu C, Sun Q, Li J (2020). CMTM6 significantly relates to PD-L1 and predicts the prognosis of gastric cancer patients. PeerJ.

[12] Guan X, Zhang C, Zhao J, Sun G, Song Q, Jia W. CMTM6 overexpression is associated with molecular and clinical characteristics of malignancy and predicts poor prognosis in gliomas. EBioMedicine. 2018;35:233–43. 10.1016/j.ebiom.2018.08.012 PMC6156716 30131308

[13] Hou X, He S, Zhang D, Yang C, Shi Y, Zhang K. Expression and clinical significance of CMTM6 in nonsmall cell lung cancer. DNA Cell Biol. 2020. Epub ahead of print. 10.1089/dna.2020.5564 33090010

[14] Wang H, Gao J, Zhang R, Li M, Peng Z, Wang H (2020). Molecular and immune characteristics for lung adenocarcinoma patients with CMTM6 overexpression. Int Immunopharmacol.

[15] Peng QH, Wang CH, Chen HM, Zhang RX, Pan ZZ, Lu ZH, et al. CMTM6 and PD-L1 coexpression is associated with an active immune microenvironment and a favorable prognosis in colorectal cancer. J Immunother Cancer. 2021;9:e001638. 10.1136/jitc-2020-001638 PMC7883863 33579737

[16] Niculescu AB, Chen X, Smeets M, Hengst L, Prives C, Reed SI (1998). Effects of p21(Cip1/Waf1) at both the G1/S and the G2/M cell cycle transitions: pRb is a critical determinant in blocking DNA replication and in preventing endoreduplication. Mol Cell Biol.

[17] Dulic V, Stein GH, Far DF, Reed SI (1998). Nuclear accumulation of p21Cip1 at the onset of mitosis: a role at the G2/M-phase transition. Mol Cell Biol.

[18] Karimian A, Ahmadi Y, Yousefi B (2016). Multiple functions of p21 in cell cycle, apoptosis and transcriptional regulation after DNA damage. DNA Repair (Amst).

[19] Chen J, Jackson PK, Kirschner MW, Dutta A (1995). Separate domains of p21 involved in the inhibition of Cdk kinase and PCNA. Nature.

[20] Luo Y, Hurwitz J, Massague J (1995). Cell-cycle inhibition by independent CDK and PCNA binding domains in p21Cip1. Nature.

[21] Blagosklonny MV, Wu GS, Omura S, el-Deiry WS (1996). Proteasome-dependent regulation of p21WAF1/CIP1 expression. Biochem Biophys Res Commun.

[22] Warfel NA, El-Deiry WS (2013). p21WAF1 and tumourigenesis: 20 years after. Curr Opin Oncol.

[23] Gupta SC, Kim JH, Prasad S, Aggarwal BB (2010). Regulation of survival, proliferation, invasion, angiogenesis, and metastasis of tumor cells through modulation of inflammatory pathways by nutraceuticals. Cancer Metastasis Rev.

[24] Abbas T, Dutta A (2009). p21 in cancer: intricate networks and multiple activities. Nat Rev Cancer.

[25] Frescas D, Pagano M (2008). Deregulated proteolysis by the F-box proteins SKP2 and beta-TrCP: tipping the scales of cancer. Nat Rev Cancer.

[26] Abbas T, Dutta A (2011). CRL4Cdt2: master coordinator of cell cycle progression and genome stability. Cell Cycle.

[27] Amador V, Ge S, Santamaria PG, Guardavaccaro D, Pagano M (2007). APC/C(Cdc20) controls the ubiquitin-mediated degradation of p21 in prometaphase. Mol Cell.

[28] Narasimha AM, Kaulich M, Shapiro GS, Choi YJ, Sicinski P, Dowdy SF. Cyclin D activates the Rb tumor suppressor by mono-phosphorylation. Elife. 2014;3:e02872. 10.7554/eLife.02872 PMC4076869 24876129

[29] Hochegger H, Takeda S, Hunt T (2008). Cyclin-dependent kinases and cell-cycle transitions: does one fit all?. Nat Rev Mol Cell Biol.

[30] Raoul JL, Forner A, Bolondi L, Cheung TT, Kloeckner R, de Baere T (2019). Updated use of TACE for hepatocellular carcinoma treatment: How and when to use it based on clinical evidence. Cancer Treat Rev.

[31] Zhu X, Qi G, Li C, Bei C, Tan C, Zhang Y (2019). Expression and clinical significance of CMTM6 in hepatocellular carcinoma. DNA Cell Biol.

[32] Huang X, Xiang L, Wang B, Hu J, Liu C, Ren A (2021). CMTM6 promotes migration, invasion, and EMT by interacting with and stabilizing vimentin in hepatocellular carcinoma cells. J Transl Med.

[33] Yugawa K, Itoh S, Yoshizumi T, Iseda N, Tomiyama T, Morinaga A (2021). CMTM6 stabilizes PD-L1 expression and is a new prognostic impact factor in hepatocellular carcinoma. Hepatol Commun.

[34] Zhang L, Chen J, Ning D, Liu Q, Wang C, Zhang Z (2019). FBXO22 promotes the development of hepatocellular carcinoma by regulating the ubiquitination and degradation of p21. J Exp Clin Cancer Res.

[35] Liu X, Zhang F, Zhang Y, Li X, Chen C, Zhou M (2018). PPM1K regulates hematopoiesis and leukemogenesis through CDC20-mediated ubiquitination of MEIS1 and p21. Cell Rep.

[36] Gartel AL, Radhakrishnan SK (2005). Lost in transcription: p21 repression, mechanisms, and consequences. Cancer Res.

[37] Giacinti C, Giordano A (2006). RB and cell cycle progression. Oncogene.

[38] Dyson N (1998). The regulation of E2F by pRB-family proteins. Genes Dev.

[39] Moldovan GL, Pfander B, Jentsch S (2007). PCNA, the maestro of the replication fork. Cell.

[40] Aasland D, Gotzinger L, Hauck L, Berte N, Meyer J, Effenberger M (2019). Temozolomide induces senescence and repression of DNA repair pathways in glioblastoma cells via activation of ATR-CHK1, p21, and NF-kappaB. Cancer Res.

[41] Wei X, Zhao L, Ren R, Ji F, Xue S, Zhang J (2021). MiR-125b loss activated HIF1alpha/pAKT loop, leading to transarterial chemoembolization resistance in hepatocellular carcinoma. Hepatol (Baltim, Md).

[42] Ding X, He M, Chan AWH, Song QX, Sze SC, Chen H (2019). Genomic and epigenomic features of primary and recurrent hepatocellular carcinomas. Gastroenterology.

