# Supplementary material for: CMTM6 inhibits tumor growth and reverses chemoresistance by preventing ubiquitination of p21 in hepatocellular carcinoma
Source: Cell Death Dis. 2022 Mar 19;13(3):251. doi: 10.1038/s41419-022-04676-1 (PMC8933468; doi:10.1038/s41419-022-04676-1)
Supplement: Supplementary file 1 — Supplementary materials and methods [file 41419_2022_4676_MOESM1_ESM.docx]

**Supplementary materials and methods**

**Cell lines**

Human HCC cell lines (HepG2, Hep3B, HuH-7, SK-Hep-1) and immortalized liver cell lines (L-02) were purchased from the Cell Bank of the Chinese Academy of Sciences (Shanghai, China). The HepG2, Hep3B and HuH-7 cells were cultured in Dulbecco's modified Eagle's medium (DMEM; Gibco, Thermo Fisher Scientific Inc., Waltham, MA, USA) supplemented with 10% fetal bovine serum (FBS; Gibco, Thermo Fisher Scientific Inc.). The L-02 and SK-Hep-1 cells were maintained in RPMI 1640 medium (Gibco, Thermo Fisher Scientific Inc.) containing 10% FBS. For the ubiquitination assay, the cells were treated with 20 μM MG132 for 6 h before harvesting. All cell lines were grown in a humidified atmosphere of 5% CO_2_ at 37 °C.

**RNA interference and lentivirus transduction**

In order to stably overexpress or knock down CMTM6 in HCC cells, recombinant lentivirus carrying a human CMTM6 overexpression plasmid, short hairpin RNA (shRNA) or the corresponding empty vectors were purchased from GenePharma Co., Ltd. (Shanghai, China). The following shRNA target sequences were used: shCMTM6-1 (5′-GAAGTTGTATCACAATGTA-3′), and shCMTM6-2 (5′-GTGCCTTTCTTCTGAGTCT-3′). Target cells were infected with lentivirus for 24 h according to the manufacturer’s instructions, followed by selection with 2 μg/mL of puromycin after 48 h. The efficiency of overexpression and knockdown was verified by qPCR and Western blot analysis. The stable cell lines were designated as HuH-7/OE-NC, HuH-7/OE-CMTM6, HepG2/shNC, HepG2/shCMTM6-1 and HepG2/shCMTM6-2.

**Quantitative real-time polymerase chain reaction (qPCR)**

Total RNA was extracted using the RNA Quick Purification Kit (ESscience) according to the manufacturer’s protocol. The reverse transcription was performed on 1 µg of total RNA using the Fast Reverse Transcription kit (ESscience). qPCR was performed to measure gene mRNA expression using the GoTaq qPCR Master Mix (Promega Corporation, Madison, WI, USA). The sequences of the primers are listed in Table S1.

**Immunohistochemistry (IHC) analysis**

The tissue sections were deparaffinized with xylene, rehydrated, antigen retrieved by microwave heating and then incubated successively with 0.3% hydrogen peroxide for 10 min and goat serum for 30 min to block endogenous peroxidase activity and nonspecific staining. Slides were incubated with the appropriate primary antibodies overnight in a humidified chamber at 4°C. After washing three times with PBS, the slides then incubated with HRP-conjugated secondary antibody using the Envision^TM^ Detection Kit (Gene Tech Co., Ltd., Shanghai. China) for 30 min. The 3, 3′-diaminobenzidine (DAB) tetrahydrochloride was used to stain the tissue sections, and the sections were then counter-stained with hematoxylin. The slides were then dehydrated, cleared, and evaluated.

The staining intensity (SI) was evaluated by multiplying the staining value (0, negative; 1, weak; 2, moderate; 3, intense) and the percentage of stained cells (0, 0%; 1, 1–25%; 2, 26–75%; 3, 75-100%). The SI levels of CMTM6 were divided into four groups: negative (0 ≤ SI < 3, 37%), low (3≤ SI <5, 35%), medium (5≤ SI <7, 19%), high (7≤ SI < 9, 9%). We defined the specimens in the negative and low groups as having low CMTM6 expression, while those in the medium and high group as having high CMTM6 expression. The scores were calculated independently by two experienced pathologists who were blinded to the clinical outcomes.

**Cell counting kit-8 (CCK-8) cell proliferation assay**

Cell suspension (100 μL/well) were seeded in 96-well plates at a density of 2 × 10^3^ cells/well. At each time point, 10 μL of CCK-8 solution (Dojindo Laboratories, Kumamoto, Japan) was added directly to each well with the test cells and incubated for 1 h at 37°C. Afterwards, the absorbance was measured at 450 nm. Each experiment was performed in triplicate.

**Bromodeoxyuridine (BrdU) incorporation assay**

The BD Pharmingen™ BrdU Flow Kit (BD Biosciences, San Jose, CA, USA) was used to evaluate DNA synthesis and proliferation rate of HCC cells according to the manufacturer’s instructions. Briefly, cells were seeded in 6-well plates at a density of 2-3×10^5^ cells per well and allowed to reach 60-70% confluence within 24 h. Then, the cells were labeled with BrdU, at a final concentration of 10 μM in cell culture medium, and incubated for 30 min. Afterwards, the cells were collected and prepared for flow cytometry according to the manufacturer’s protocol.

**Colony formation assay**

Cells were seeded in 6-well plates at a density of 500 cells per well and incubated for 10 days at 37°C in a humidified chamber with 5% CO_2_. After fixation with 4% paraformaldehyde for 10 min, the colonies were stained with 1% crystal violet for 10 min. The stained colonies were counted, and the colony forming efficiency (CFE %) was calculated using the following formula: CFE % = (colony number/plated cell number) × 100. This experiment was performed in triplicate.

**Cell cycle analysis**

Cells were harvested, washed twice with cold PBS and fixed in cold 75% ethanol at -20 °C overnight. The fixed cells were washed twice with cold PBS and then incubated with 0.5 mL of propidium iodide (PI)/RNase Staining Buffer (BD Biosciences) for 15 min at room temperature in the dark. The cell cycle distribution was analyzed by ﬂow cytometry on a NovoCyte^®^ Flow Cytometer System (ACEA Biosciences Inc., San Diego, CA, USA). All experiments were performed in triplicate.

**Apoptosis analysis**

Cell apoptosis was analyzed by flow cytometry using the Annexin V-APC/7-AAD Apoptosis kits (ESscience) according to the manufacturer’s protocol. Briefly, after exposure to the different treatments, cells were harvested and suspended in 100 μL binding buffer (1×). Then, 5 μL of Annexin V-APC and 10 μL of 7-AAD were added to each sample well. After incubation for 15 min in the dark, the stained cells were analyzed by flow cytometry within 1 h.

**Dual-luciferase reporter assay**

The indicated cells were seeded in 96-well plates and cotransfected with pcDNA3.1-E2F1 and pRL-TK as an internal control and pGL4.10-target genes-promoter (PCNA, TK1, CDC6). After 48 hours, the cells were harvested and use to prepare the cell lysates, which were used to assess the luciferase activity using the dual-luciferase reporter assay according to the manufacturer’s instructions (Promega Corporation). Then, the relative luciferase activity was determined (Firefly luciferase/Renilla luciferase).

**Xenograft model**

Female 4-week old NCG mice were purchased from the GemPharmatech Co., Ltd. (Nanjing, China). The mice were subcutaneously injected with a total of 3×10^6^ tumor cells suspended in 100 μL of PBS containing 50% Matrigel Basement Membrane Matrix (BD Biosciences) and then randomly assigned to 5 groups of 5 mice per group. Tumor volume was measured every 3 days, and tumor growth was calculated using the following formula: 0.52 × larger diameter × (small diameter)^2^. All mice were euthanized at 4 weeks after inoculation and the tumors were harvested, photographed, weighed and then embedded in paraffin. Serial 4.0 mm sections were obtained and analyzed by histological analysis after staining with hematoxylin and eosin (H&E) and IHC analysis with the appropriate primary antibodies. This study protocol was approved by the Animal Care and Use Committee of the Sun Yat-Sen University Cancer Center, Sun Yat-Sen University.
