# Supplementary material for: CMTM6 inhibits tumor growth and reverses chemoresistance by preventing ubiquitination of p21 in hepatocellular carcinoma
Source: Cell Death Dis. 2022 Mar 19;13(3):251. doi: 10.1038/s41419-022-04676-1 (PMC8933468; doi:10.1038/s41419-022-04676-1)
Supplement: Supplementary file 2 — Supplemental Tables [file 41419_2022_4676_MOESM2_ESM.docx]

| Table S1. The sequences of the primers used in this research | | |
| --- | --- | --- |
| Primer |  | 5’-3’ Sequence |
| CMTM6 | F | TTTCCACACATGACAGGACTTC |
|  | R | GGCTTCAGCCCTAGTGGTAT |
| CDKN1A | F | CGATGGAACTTCGACTTTGTCA |
|  | R | GCACAAGGGTACAAGACAGTG |
| β-actin | F | CATGTACGTTGCTATCCAGGC |
|  | R | CTCCTTAATGTCACGCACGAT |
| GAPDH | F | GGAGCGAGATCCCTCCAAAAT |
|  | R | GGCTGTTGTCATACTTCTCATGG |
| PCNA | F | GCGTGAACCTCACCAGTATGT |
|  | R | TCTTCGGCCCTTAGTGTAATGAT |
| CDC6 | F | CCAGGCACAGGCTACAATCAG |
|  | R | AACAGGTTACGGTTTGGACATT |
| TK1 | F | GCCAAAGACACTCGCTACAG |
|  | R | CCCCTCGTCGATGCCTATG |
| POLA1 | F | AGAAGCTCGCAGTGACAAAAC |
|  | R | AGGTGGTGGAGTTATTTGAGGT |
| ORC1 | F | ACTACCCCACAAGGCTGAAGA |
|  | R | AGTGCAGTTTTCGATCCAACA |
| ORC3 | F | CCTCCTGTTGTCGTTATCTTGAA |
|  | R | TCATGGAGATGTTGACTGCTGA |
| cyclin E1 | F | GCCAGCCTTGGGACAATAATG |
|  | R | CTTGCACGTTGAGTTTGGGT |
| cyclin A1 | F | ACATGGATGAACTAGAGCAGGG |
|  | R | GAGTGTGCCGGTGTCTACTT |
| CDC45 | F | CTTGAAGTTCCCGCCTATGAAG |
|  | R | GCATGGTTTGCTCCACTATCTC |
| CDT1 | F | GACATGATGCGTAGGCGTTT |
|  | R | GGCTCGATGTCGGGTACTTC |
| CDC25A | F | GTGAAGGCGCTATTTGGCG |
|  | R | TGGTTGCTCATAATCACTGCC |
| CDK1 | F | AAACTACAGGTCAAGTGGTAGCC |
|  | R | TCCTGCATAAGCACATCCTGA |
| DHFR | F | TTCCAGAGAATGACCACAACCTC |
|  | R | CTCCTTGTGGAGGTTCCTTGA |
| SOX2 | F | TACAGCATGTCCTACTCGCAG |
|  | R | GAGGAAGAGGTAACCACAGGG |
| NANOG | F | TCTGGACACTGGCTGAATCCT |
|  | R | CGCTGATTAGGCTCCAACCAT |
| PROM1 | F | GGCCCAGTACAACACTACCAA |
|  | R | ATTCCGCCTCCTAGCACTGAA |
| MYC | F | GTCAAGAGGCGAACACACAAC |
|  | R | TTGGACGGACAGGATGTATGC |
| POU5F1 | F | CTTGAATCCCGAATGGAAAGGG |
|  | R | CCTTCCCAAATAGAACCCCCA |
| BMI1 | F | TGGACTGACAAATGCTGGAGA |
|  | R | GAAGATTGGTGGTTACCGCTG |

| Table S2. The primary antibodies used in this research | | | | |
| --- | --- | --- | --- | --- |
| Name of the antibody | Catalog number | Application | Dilution | Brand of the antibody |
| anti-CMTM6 Rabbit pAb | HPA026980 | WB | 1:500 | Sigma-Aldrich |
|  |  | IHC | 1:20 |  |
|  |  | IP | 1:50 |  |
| anti-CMTM6 Rabbit mAb | 34557 | WB | 1:1000 | Cell Signaling Technology |
| anti-p21 Rabbit mAb | 2947 | WB | 1:1000 | Cell Signaling Technology |
|  |  | IHC | 1:50 |  |
| anti-p21 Mouse mAb | 2946 | WB | 1:2000 | Cell Signaling Technology |
|  |  | IP | 1:500 |  |
| anti-p53 Rabbit mAb | 2524 | WB | 1:1000 | Cell Signaling Technology |
| anti-β-actin Mouse mAb | 66009-1-Ig | WB | 1:5000 | Proteintech |
| anti-Ki67 Rabbit mAb | ab15580 | IHC |  | Abcam |
| anti-Ubiquitin Mouse mAb | 3936 | WB | 1:1000 | Cell Signaling Technology |
| anti-Rb Mouse mAb | 9309 | WB | 1:1000 | Cell Signaling Technology |
| anti- phospho-Rb Rabbit mAb | 8516 | WB | 1:1000 | Cell Signaling Technology |
| anti-SKP2 Rabbit mAb | 2652 | WB | 1:1000 | Cell Signaling Technology |
| anti-CDT2 (DTL) Rabbit pAb | 12896-1-AP | WB | 1:1000 | Proteintech |
| anti-CDC20 Rabbit mAb | 14866 | WB | 1:1000 | Cell Signaling Technology |
| anti-Flag M2 Mouse mAb | F1804 | IP | 1:250 | Sigma-Aldrich |
| anti-Myc Mouse mAb | M4439 | IP | 1:500 | Sigma-Aldrich |

| Table S3. Association between CMTM6 expression and the clinicopathological features of HCC | | | | | |
| --- | --- | --- | --- | --- | --- |
| Clinicopathologic variables |  | CMTM6 expression | | *X^2^* | *P* value |
|  |  | low | high |  |  |
| All case |  | 122 | 45 |  |  |
| Gender | Male | 113 | 39 | 1.427 | 0.232 |
|  | Female | 9 | 6 |  |  |
| Age | ≤50 | 60 | 23 | 0.049 | 0.825 |
|  | >50 | 62 | 22 |  |  |
| HBsAg | Positive | 110 | 38 | 1.067 | 0.302 |
|  | Negative | 12 | 7 |  |  |
| HCV Ag | Negative | 120 | 43 | 1.106 | 0.293 |
|  | Positive | 2 | 2 |  |  |
| AFP (ng/mL) | ≤400 | 68 | 30 | 1.619 | 0.203 |
|  | >400 | 54 | 15 |  |  |
| ALT (U/L) | ≤40 | 72 | 23 | 0.838 | 0.360 |
|  | >40 | 50 | 22 |  |  |
| AST (U/L) | ≤40 | 64 | 24 | 0.010 | 0.920 |
|  | >40 | 58 | 21 |  |  |
| TBIL (μmol/L) | ≤21 | 100 | 42 | 3.336 | 0.068 |
|  | >21 | 22 | 3 |  |  |
| ALB (g/L) | ≥35 | 119 | 43 | 0.446 | 0.504 |
|  | <35 | 3 | 2 |  |  |
| Cirrhosis | Negative | 79 | 29 | 0.001 | 0.970 |
|  | Positive | 43 | 16 |  |  |
| Tumor size(cm) | ≤5 | 42 | 24 | 4.917 | 0.027 |
|  | >5 | 80 | 21 |  |  |
| Tumor number | single | 85 | 38 | 3.697 | 0.055 |
|  | multiple | 37 | 7 |  |  |
| PVTT | Negative | 116 | 45 | 2.296 | 0.130 |
|  | Portal vein | 6 | 0 |  |  |
| MVI | Negative | 104 | 43 | 3.315 | 0.069 |
|  | Positive | 18 | 2 |  |  |
| Satellite lesions | Negative | 86 | 39 | 4.569 | 0.033 |
|  | Positive | 36 | 6 |  |  |
| Lymphatic transfer | Negative | 120 | 45 | 0.747 | 0.388 |
|  | Positive | 2 | 0 |  |  |
| BCLC stage | 0+A | 100 | 43 | 4.933 | 0.026 |
|  | B+C | 22 | 2 |  |  |
| TNM stage | I+II | 90 | 40 | 4.357 | 0.037 |
|  | III+IV | 32 | 5 |  |  |
| Postoperative recurrence | Negative | 57 | 30 | 5.240 | 0.022 |
|  | Positive | 65 | 15 |  |  |

MVI: Microvascular invasion; PVTT: portal vein tumor thrombus;

| Table S4. Univariate and multivariate analyses of factors associated with overall survival. | | | | | | |
| --- | --- | --- | --- | --- | --- | --- |
|  | Univariate analysis | | | Multivariate analysis | | |
|  | HR | 95%CI | *P* value | HR | 95%CI | *P* value |
| CMTM6 expression | 0.738 | 0.652 ~ 0.834 | 0.000 | 0.822 | 0.720 ~ 0.939 | 0.004 |
| Gender | 0.972 | 0.446 ~ 2.119 | 0.943 |  |  |  |
| Age | 1.284 | 0.806 ~ 2.045 | 0.292 |  |  |  |
| HBsAg | 0.798 | 0.397 ~ 1.606 | 0.528 |  |  |  |
| AFP | 1.476 | 0.930 ~ 2.342 | 0.098 |  |  |  |
| HCV Ag | 0.720 | 0.100 ~ 5.186 | 0.745 |  |  |  |
| Cirrhosis | 1.617 | 1.015 ~ 2.576 | 0.043 | 2.289 | 1.405 ~ 3.730 | 0.001 |
| Tumor size | 1.154 | 1.089 ~ 1.222 | 0.000 | 1.120 | 1.042 ~ 1.203 | 0.002 |
| Tumor number | 4.523 | 2.839 ~ 7.205 | 0.000 | 2.713 | 0.647 ~ 11.369 | 0.172 |
| Satellite lesions | 4.578 | 2.866 ~ 7.315 | 0.000 | 1.673 | 0.396 ~ 7.062 | 0.484 |
| MVI | 2.822 | 1.544 ~ 5.157 | 0.001 | 1.545 | 0.776 ~ 3.073 | 0.215 |
| PVTT | 1.070 | 0.336 ~ 3.403 | 0.909 |  |  |  |
| Lymphatic transfer | 3.183 | 0.774 ~ 13.096 | 0.109 |  |  |  |

| Table S5. Univariate and multivariate analyses of factors associated with disease free survival. | | | | | | |
| --- | --- | --- | --- | --- | --- | --- |
|  | Univariate analysis | | | Multivariate analysis | | |
|  | HR | 95%CI | *P* value | HR | 95%CI | *P* value |
| CMTM6 expression | 0.808 | 0.723 ~ 0.903 | 0.000 | 0.868 | 0.773~0.975 | 0.017 |
| Gender | 0.602 | 0.244 ~ 1.490 | 0.272 |  |  |  |
| Age | 1.088 | 0.701 ~ 1.689 | 0.707 |  |  |  |
| HBsAg | 1.048 | 0.504 ~ 2.179 | 0.900 |  |  |  |
| AFP | 1.191 | 0.762 ~ 1.861 | 0.442 |  |  |  |
| HCV Ag | 0.690 | 0.096 ~ 4.969 | 0.712 |  |  |  |
| Cirrhosis | 1.386 | 0.882 ~ 2.177 | 0.157 |  |  |  |
| Tumor size | 1.107 | 1.046 ~ 1.172 | 0.000 | 1.083 | 1.015 ~ 1.156 | 0.016 |
| Tumor number | 3.883 | 2.470 ~ 6.103 | 0.000 | 4.229 | 1.009 ~ 17.715 | 0.049 |
| Satellite lesions | 3.698 | 2.346 ~ 5.829 | 0.000 | 0.837 | 0.199 ~ 3.528 | 0.809 |
| MVI | 1.877 | 0.992 ~ 3.551 | 0.053 |  |  |  |
| PVTT | 1.404 | 0.513 ~ 3.845 | 0.509 |  |  |  |
| Lymphatic transfer | 4.835 | 1.165 ~ 20.069 | 0.030 | 2.156 | 0.491 ~ 9.461 | 0.308 |

| Table S6. Univariate and multivariate analyses of factors associated with survival after first TACE. | | | | | | |
| --- | --- | --- | --- | --- | --- | --- |
|  | Univariate analysis | | | Multivariate analysis | | |
|  | HR | 95%CI | *P* value | HR | 95%CI | *P* value |
| CMTM6 expression | 0.194 | 0.067 ~ 0.564 | 0.003 | 0.246 | 0.075 ~ 0.799 | 0.020 |
| Gender | 1.404 | 0.535 ~ 3.689 | 0.491 |  |  |  |
| Age | 1.266 | 0.646 ~ 2.481 | 0.492 |  |  |  |
| HBsAg | 0.304 | 0.040 ~ 2.340 | 0.253 |  |  |  |
| AFP | 0.746 | 0.362 ~ 1.537 | 0.427 |  |  |  |
| HCV Ag | 0.045 | 0.000 ~ 67.027 | 0.406 |  |  |  |
| Cirrhosis | 0.691 | 0.339 ~ 1.408 | 0.308 |  |  |  |
| Tumor size | 1.060 | 0.953 ~ 1.179 | 0.285 |  |  |  |
| Tumor number | 2.844 | 1.431 ~ 5.653 | 0.003 | 0.426 | 0.052 ~ 3.487 | 0.427 |
| Satellite lesions | 3.359 | 1.659 ~ 6.802 | 0.001 | 3.903 | 0.484 ~ 31.490 | 0.201 |
| MVI | 7.053 | 2.589 ~ 19.220 | 0.000 | 4.470 | 1.603 ~ 12.461 | 0.004 |
| PVTT | 0.644 | 0.087 ~ 4.775 | 0.667 |  |  |  |
