# Supplementary material for: CMTM6 inhibits tumor growth and reverses chemoresistance by preventing ubiquitination of p21 in hepatocellular carcinoma
Source: Cell Death Dis. 2022 Mar 19;13(3):251. doi: 10.1038/s41419-022-04676-1 (PMC8933468; doi:10.1038/s41419-022-04676-1)
Supplement: Supplementary file 3 — Supplemental figure legends [file 41419_2022_4676_MOESM3_ESM.docx]

**Supplemental figure legends**

**Figure. S1** (A) The mRNA and (B) protein expressions were detected in a normal human liver cell line (L-02) and four HCC cell lines (HepG2, Hep3B, HuH-7, SK-Hep-1). β-actin was used as a loading control. *P < 0.05.

**Figure. S2** (A) Western blot and (B) qPCR analysis results validated the regulation of CMTM6 in SK-Hep-1 and Hep3B. (C) CCK-8 assays showing the cell growth of the indicated HCC cells. (D) Colony fomarion assays of the indicated cells. (E) Evaluation of the effects of CMTM6 on HCC cells proliferation by BrdU incorporation analysis. (F and G) Effects of CMTM6 on cell spontaneous apoptosis as assessed by flow cytometric analysis. The error bars represent the mean ± SD. *** P < 0.001; ****P < 0.0001; ns means non-significant.

**Figure. S3** CMTM6 had no significant effect on the stemness of HCC. (A) Sphere formation assay. (B) Anlysis of the LIHC datasets from TCGA database showed no significant correlation between CMTM6 and the stemness-associated markers, including SOX2, NANOG, PROM1, MYC, POU5F1 and BMI1. (C) qPCR analysis for the mRNA expression of SOX2, NANOG, PROM1, MYC, POU5F1 and BMI1 in the indicated cells. The error bars represent the mean ± SD. *P < 0.5; ns means non-significant.

**Figure. S4** Representative images of H&E and IHC staining with anti-CMTM6 and anti-Ki67 on tumors of the tested mice. The images are shown at 40× and 200× magnification.

**Figure. S5** (A) Cell cycle distribution of the indicated HCC cells as assessed by flow cytometric analysis. (B) 293T cells transfected with plasmids expressing Flag-CMTM6 were subjected to immunoprecipitation with control IgG or anti-Flag antibody. The immunoprecipitates were then probed with anti-PCNA antibody. (C) 293T cells transfected with plasmids expressing Myc-p21 were subjected to immunoprecipitation with control IgG or anti-Myc antibody. The immunoprecipitates were then probed with anti-PCNA antibody. (D) qPCR analysis of CMTM6 and p21 expression in the indicated cells. (E) Typical IHC images of p21 expression status in HCC tissues. (F) qPCR analysis and (G) the LIHC datasets from TCGA database showed no significant correlation between CMTM6 and p21 mRNA expression. *P < 0.05; **P < 0.01; ns means non-significant.

**Figure. S6** p21 suppresses the proliferation of HCC cells. (A) Western blot results validated the regulation of p21 in the indicated cells. (B) Evaluation of the effects of p21 on HCC cells growth by CCK8 assays. (C) BrdU incorporation analysis showing the cell proliferation of the indicated cells. (D) Effects of p21 on cell spontaneous apoptosis as assessed by flow cytometric analysis. (E) Cell cycle distribution of the indicated HCC cells. The error bars represent the mean ± SD. *P < 0.05; **P < 0.01; ***P < 0.001; ****P < 0.0001; ns means non-significant.

**Figure. S7** (A) The indicated cells were synchronized using a double-thymidine block and released at the indicated phase. Cells were collected and stained with propidium iodide for flow cytometric analysis. (B) Western blot analysis of CMTM6 and p21 expression in the indicated cells collected in each phase. (C-E) HuH-7 and HepG2 transfected with the indicated constructs for 72 h and the levels of p21 and CMTM6 were analyzed by Western blotting. (F)Western blot analysis of SKP2, CDT2 and CDC20 expression in the indicated cells. (G) HuH-7 and HepG2 cells stably overexpressing p21 were transfected with the indicated constructs for 72h and then subjected to immunoprecipitation with anti-p21 antibody.

**Figure. S8** CMTM6 sensitizes HuH-7 and HepG2 cells to DDP. (A) The indicated cells were treated with 10 μM DDP for 0, 8 or 16 h. Cell lysates were then extracted and subjected to Western blot analysis. (B) HuH-7 and (C) HepG2 cells transfected with the indicated constructs were treated with DDP at different concentrations for 48 h and cell viability was then measured by the CCK-8 assay. (D) HuH-7 and (E) HepG2 cells transfected with the indicated constructs were treated with 10 μM DDP for different durations and cell viability was measured by the CCK-8 assay. (F) HuH-7 (top) and HepG2 (bottom) cells transfected with the indicated constructs were treated with 10 μM DDP for 48 h and cell apoptosis was assessed by flow cytometric analysis. The experiments were performed in triplicate. The error bars represent the mean ± SD. **P < 0.01; ***P < 0.001; ****P < 0.0001.

**Figure. S9** CMTM6 sensitizes SK-Hep-1 and Hep3B cells to DDP. (A and B) SK-Hep-1 and Hep3B cells transfected with the indicated constructs were treated with Dox (A) and DDP (B) at different concentrations for 48 h and cell viability was then measured by the CCK-8 assay. (C) SK-Hep-1 cells transfected with the indicated constructs were treated with 0.5 μM Dox for different durations; (D) Hep3B cells transfected with the indicated constructs were treated with 1 μM Dox for different durations; cell viability was measured by the CCK-8 assay. (E) SK-Hep-1 (top) and Hep3B (bottom) cells transfected with the indicated constructs were treated with 0.5 μM Dox for 48 h and cell apoptosis was assessed by flow cytometric analysis. (F) SK-Hep-1 and (G) Hep3B cells transfected with the indicated constructs were treated with 10 μM DDP for different durations and cell viability was measured by the CCK-8 assay. (H) SK-Hep-1 (top) and Hep3B (bottom) cells transfected with the indicated constructs were treated with 5 μM DDP for 48 h and cell apoptosis was assessed by flow cytometric analysis. The error bars represent the mean ± SD. *P < 0.05; **P < 0.01; ***P < 0.001; ****P < 0.0001.
