# Supplementary figures and images for: CMTM6 inhibits tumor growth and reverses chemoresistance by preventing ubiquitination of p21 in hepatocellular carcinoma
Source: Cell Death Dis. 2022 Mar 19;13(3):251. doi: 10.1038/s41419-022-04676-1 (PMC8933468; doi:10.1038/s41419-022-04676-1)

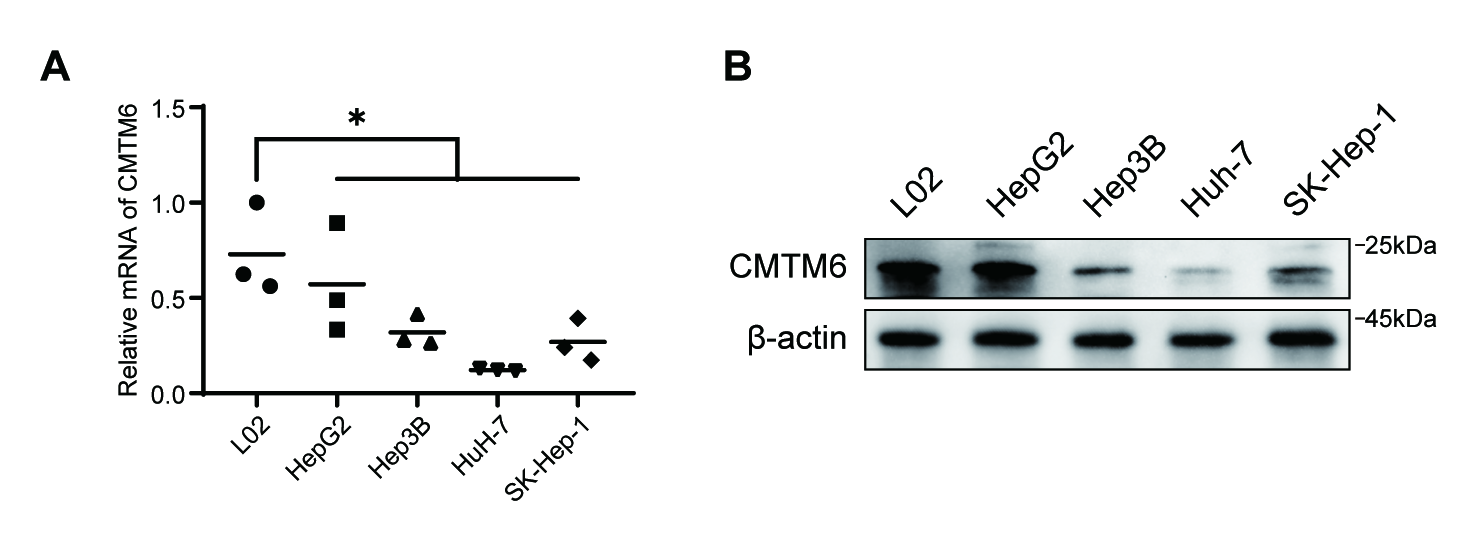

Supplement: Supplementary file 4 — Figure S1 [file 41419_2022_4676_MOESM4_ESM.tif]

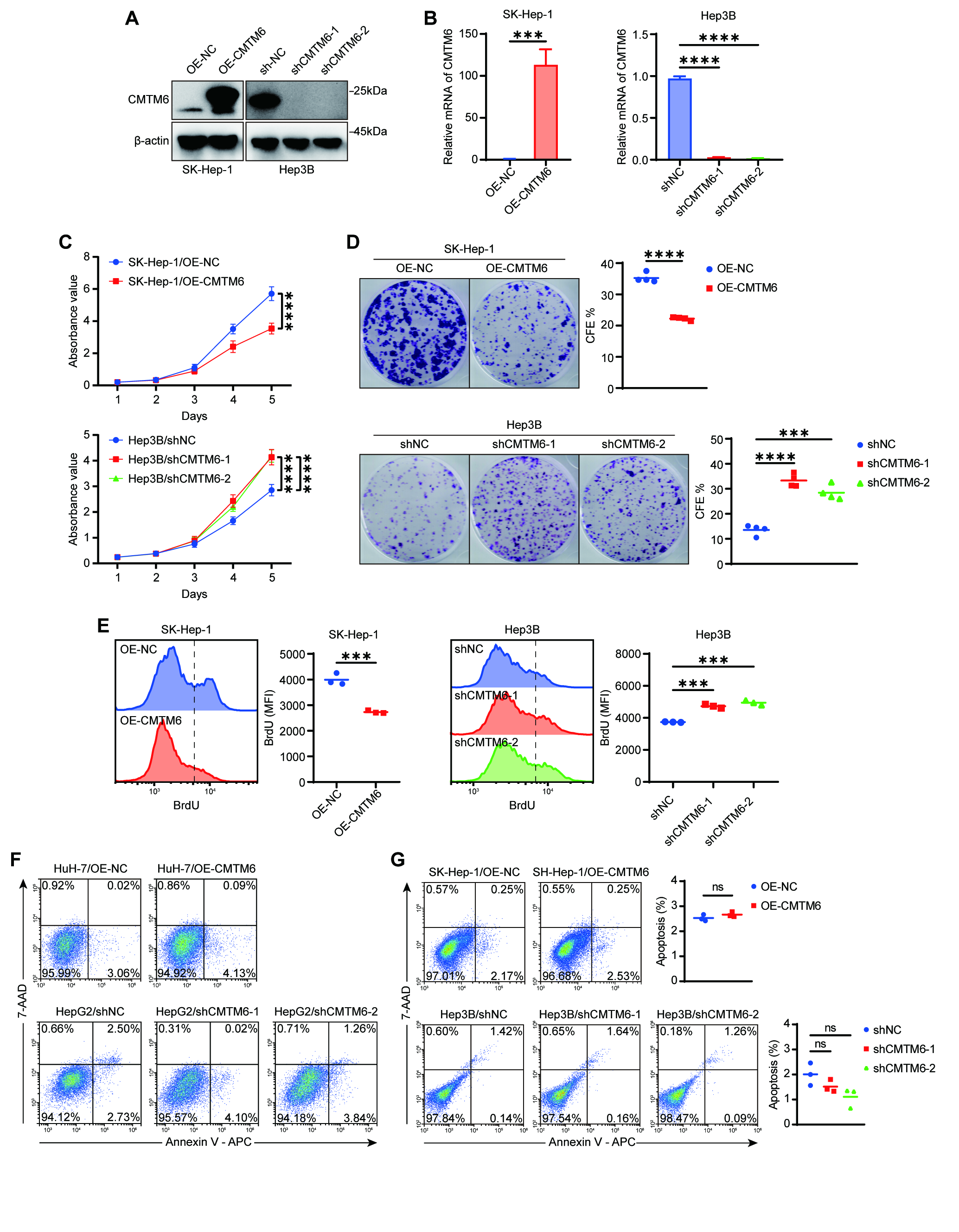

Supplement: Supplementary file 5 — Figure S2 [file 41419_2022_4676_MOESM5_ESM.tif]

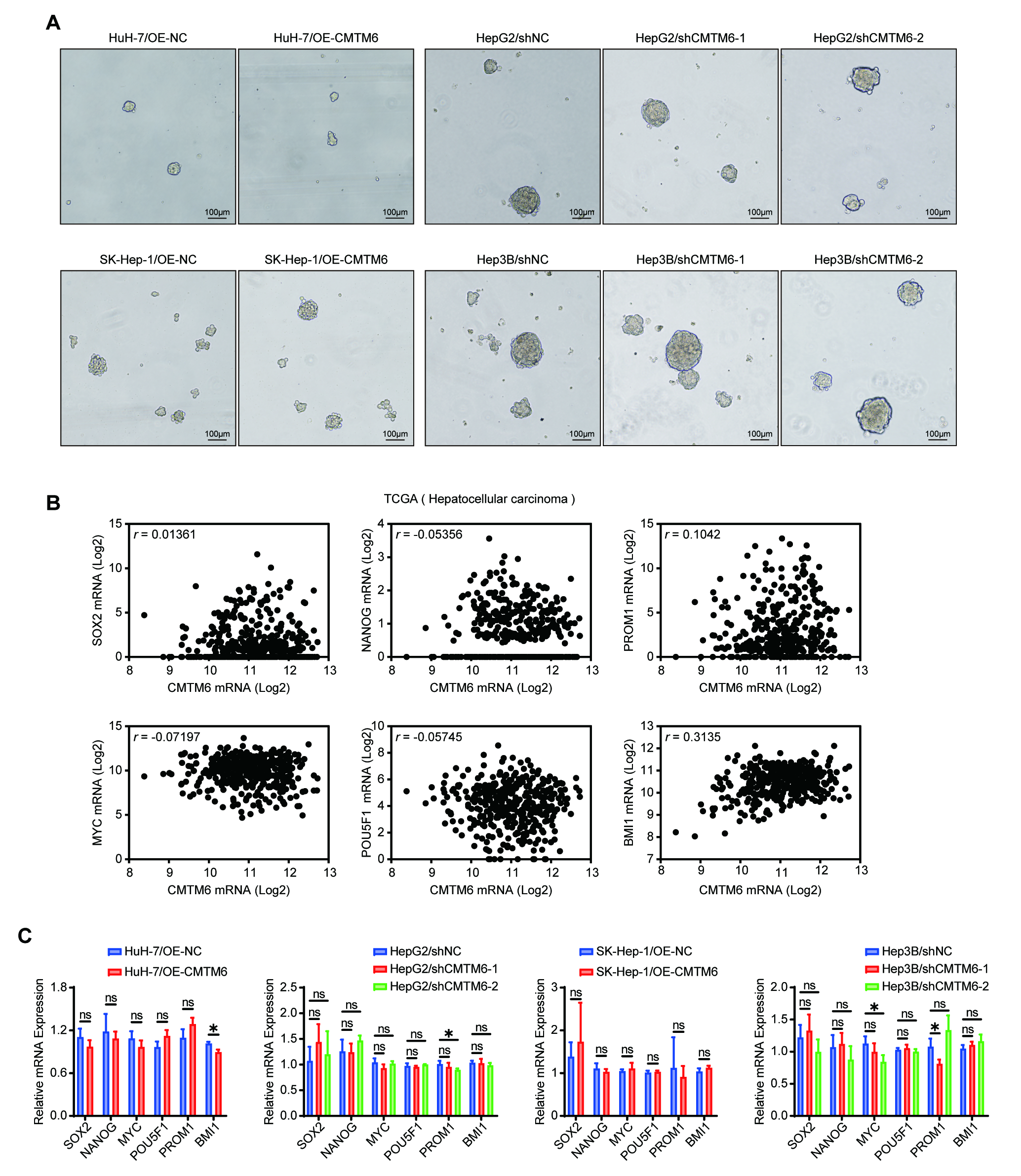

Supplement: Supplementary file 6 — Figure S3 [file 41419_2022_4676_MOESM6_ESM.tif]

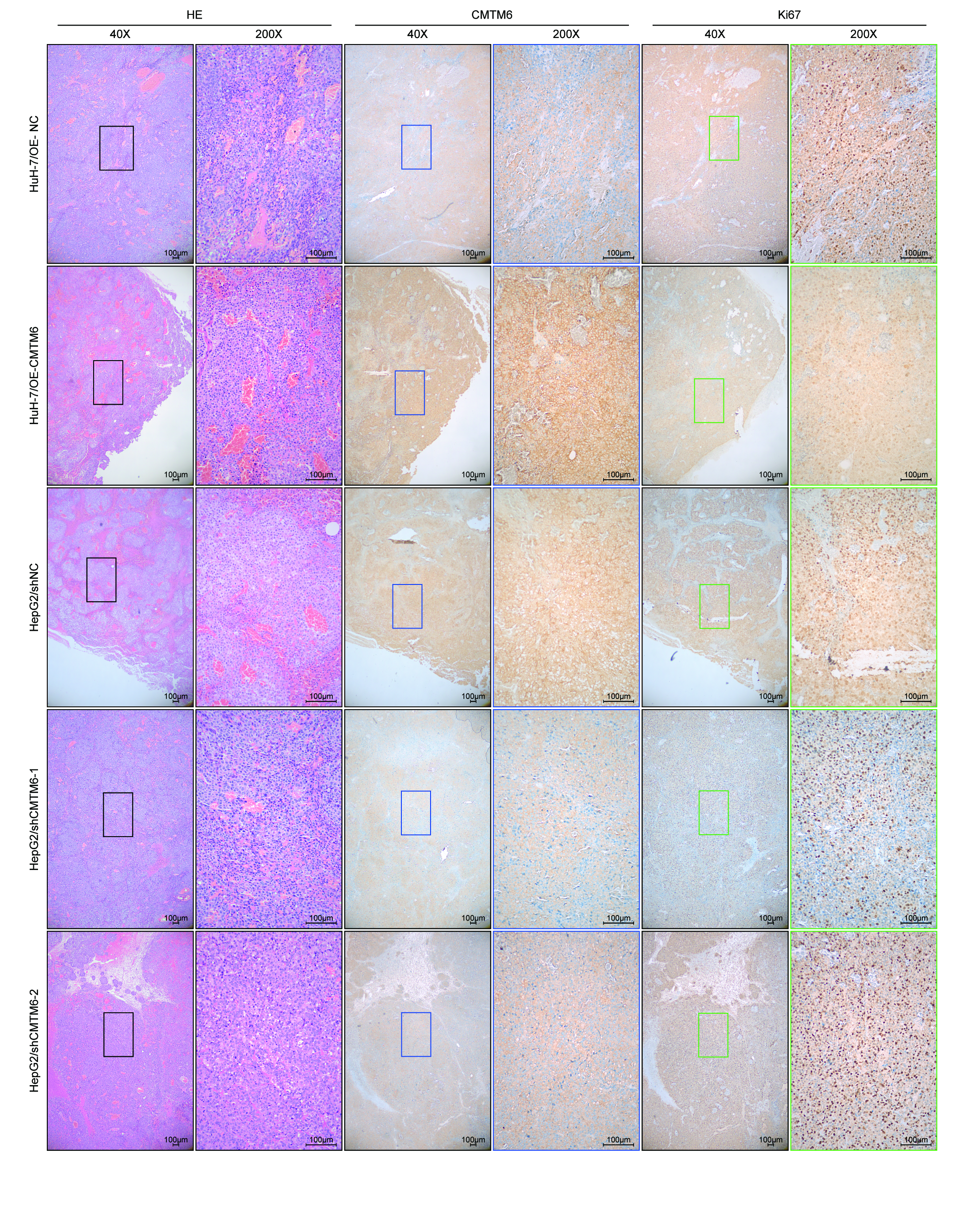

Supplement: Supplementary file 7 — Figure S4 [file 41419_2022_4676_MOESM7_ESM.tif]

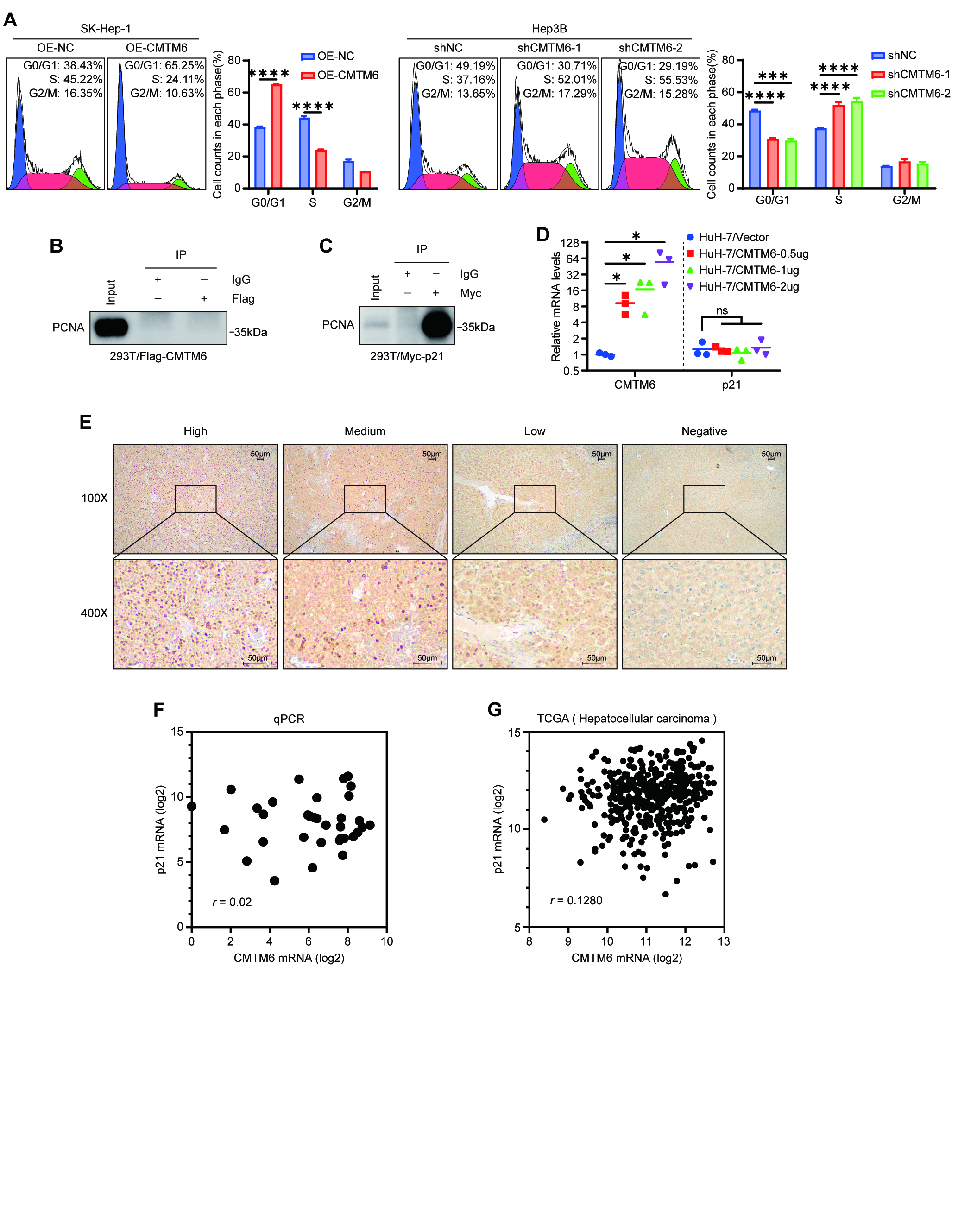

Supplement: Supplementary file 8 — Figure S5 [file 41419_2022_4676_MOESM8_ESM.tif]

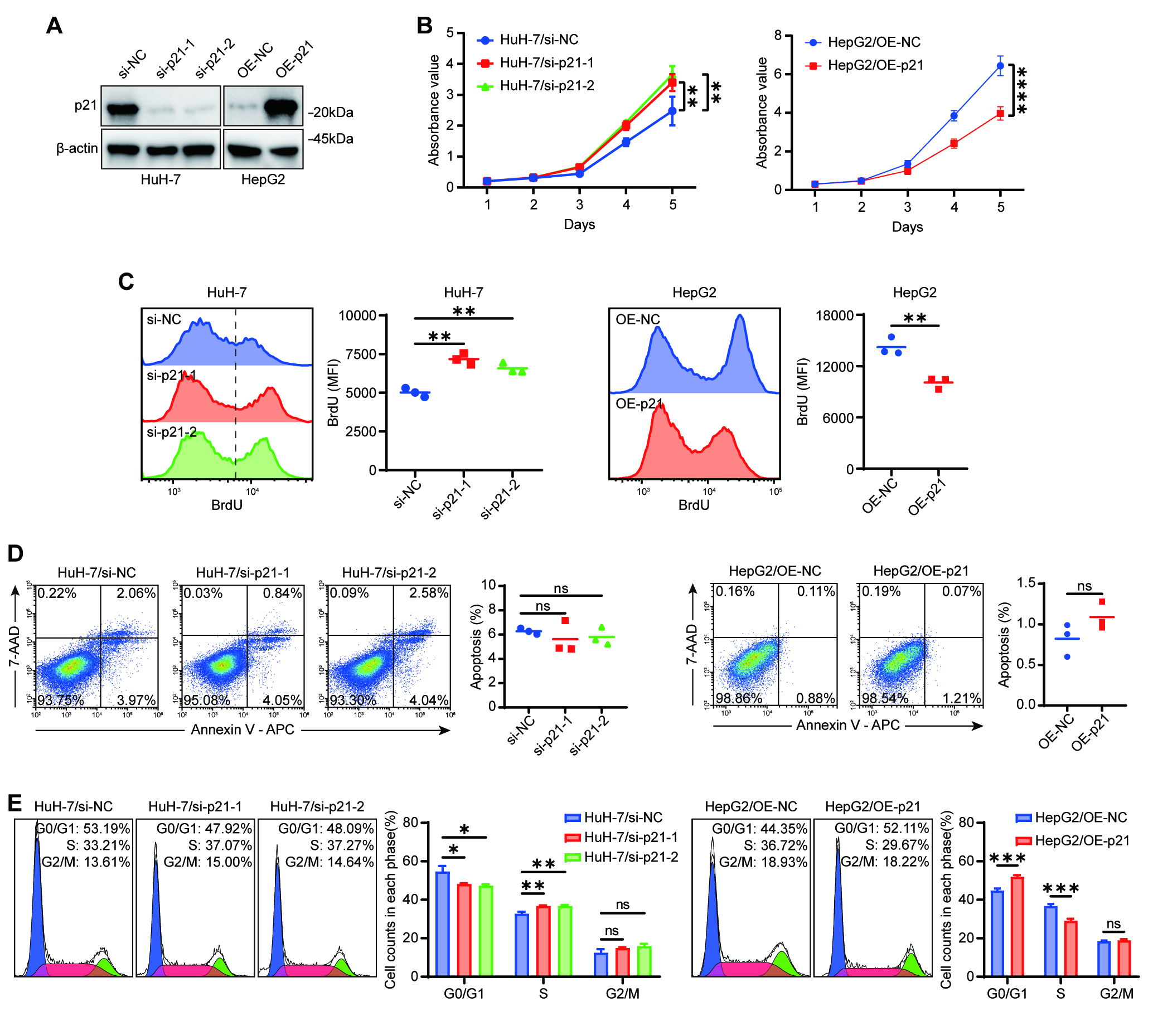

Supplement: Supplementary file 9 — Figure S6 [file 41419_2022_4676_MOESM9_ESM.tif]

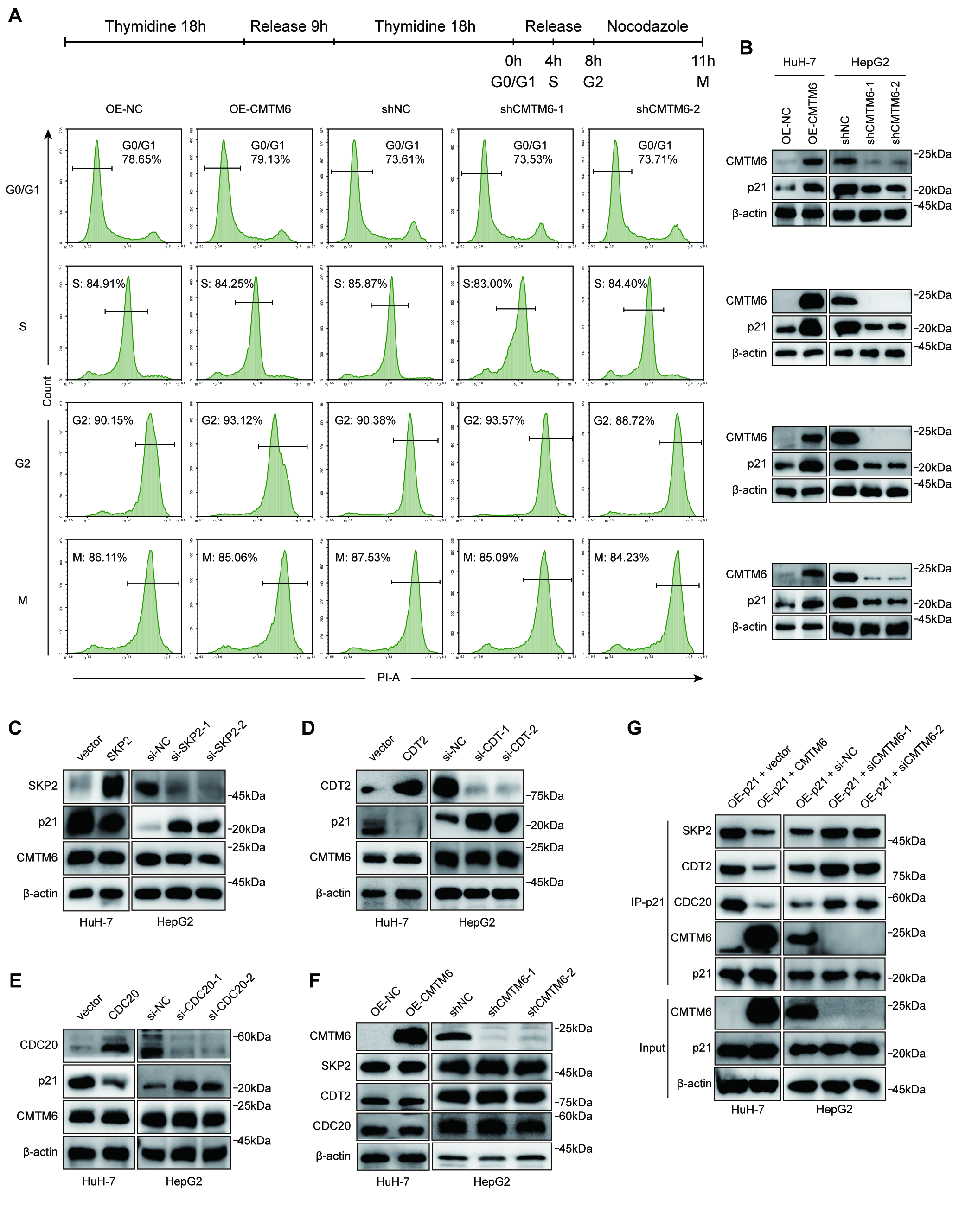

Supplement: Supplementary file 10 — Figure S7 [file 41419_2022_4676_MOESM10_ESM.tif]

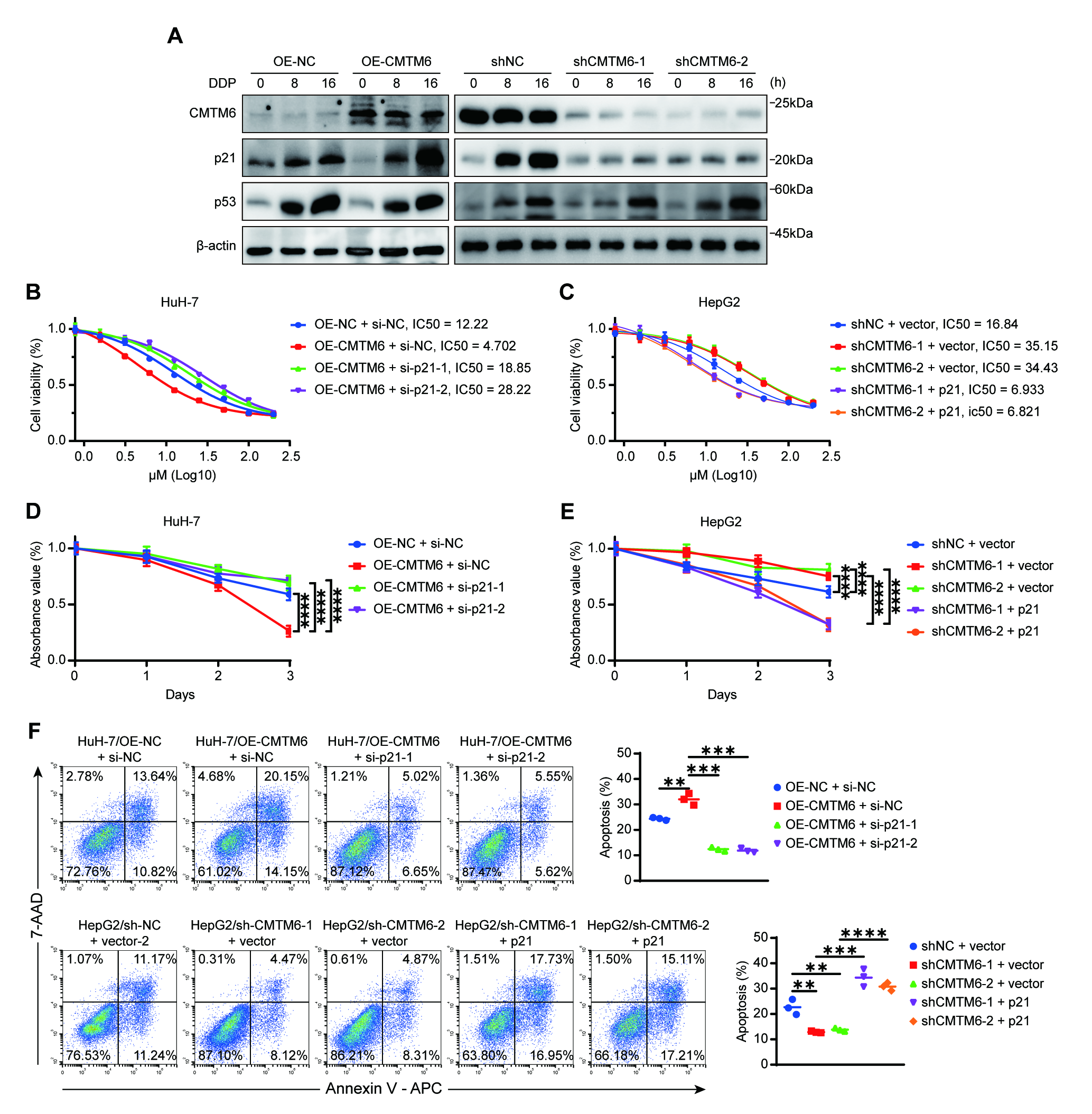

Supplement: Supplementary file 11 — Figure S8 [file 41419_2022_4676_MOESM11_ESM.tif]

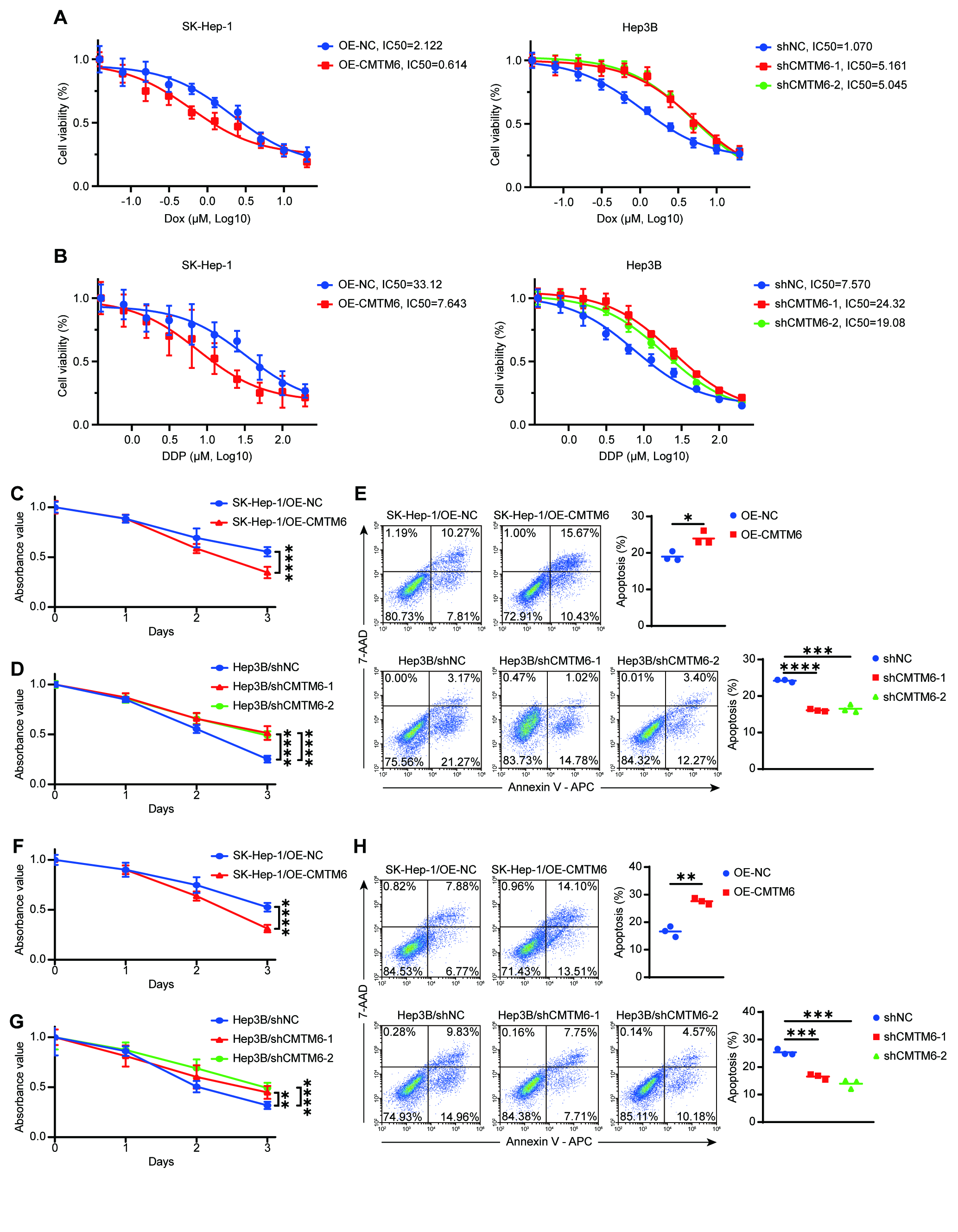

Supplement: Supplementary file 12 — Figure S9 [file 41419_2022_4676_MOESM12_ESM.tif]
