# Supplementary material for: CMTM6 inhibits tumor growth and reverses chemoresistance by preventing ubiquitination of p21 in hepatocellular carcinoma
Source: Cell Death Dis. 2022 Mar 19;13(3):251. doi: 10.1038/s41419-022-04676-1 (PMC8933468; doi:10.1038/s41419-022-04676-1)
Supplement: Supplementary file 13 — uncropped western blots [file 41419_2022_4676_MOESM13_ESM.pdf]

Figure. 1

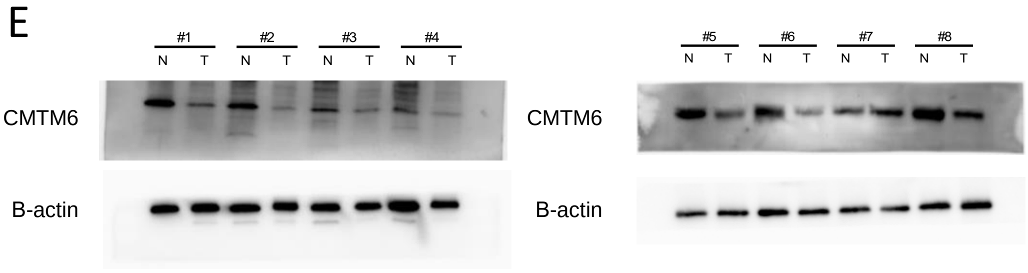

Figure. 2

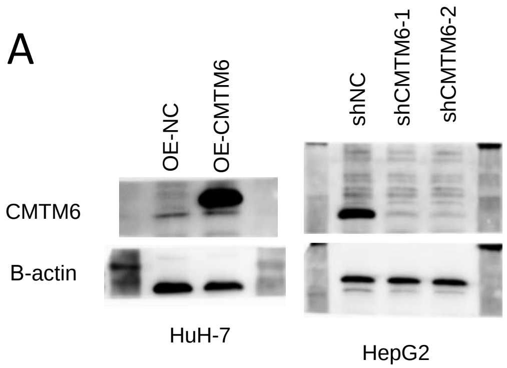

Figure. 3

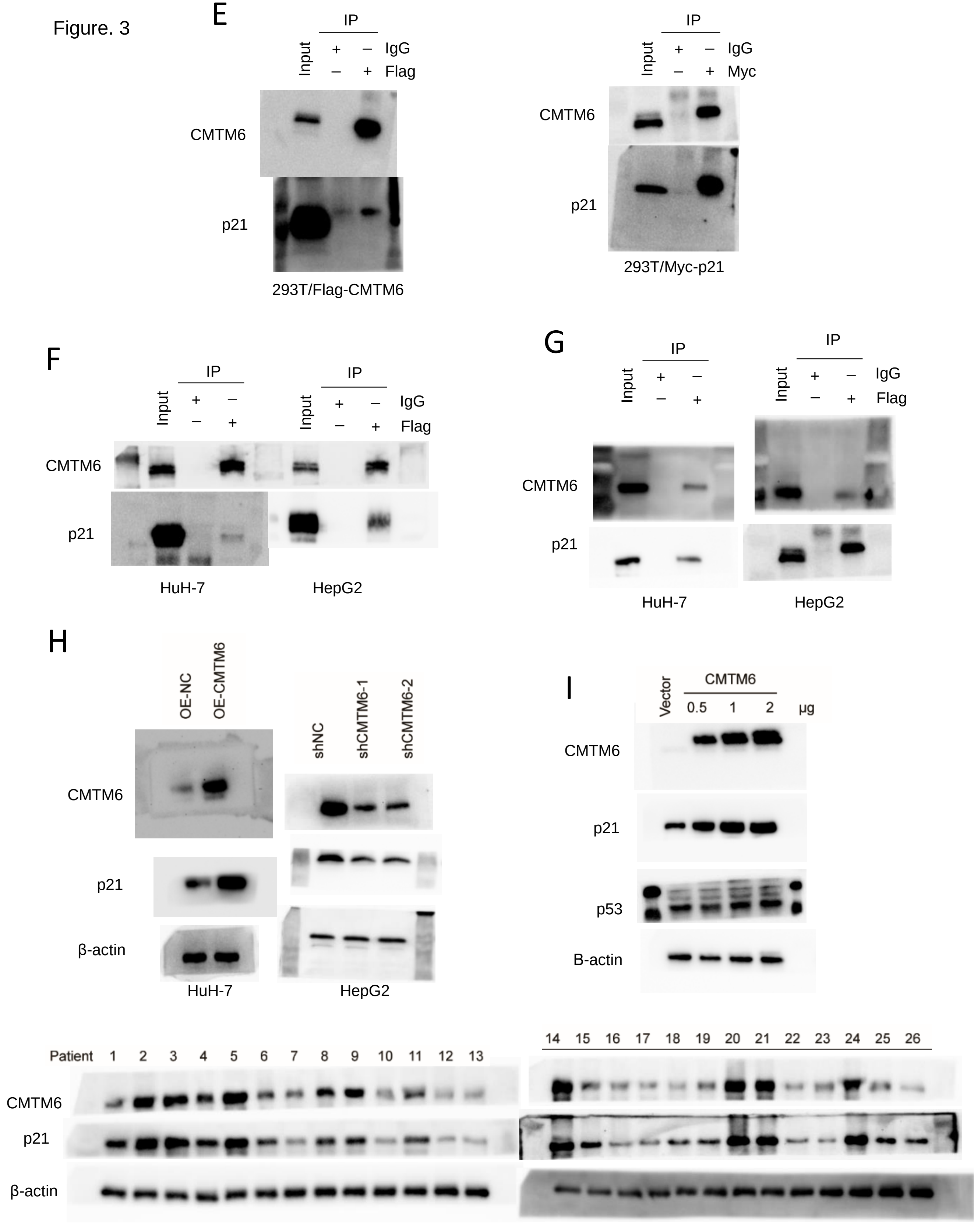

E
IP
Input
+ – IgG
– + Flag
CMTM6
p21
293T/Flag-CMTM6
IP
Input
+ – IgG
– + Myc
CMTM6
p21
293T/Myc-p21
F
IP
Input
+ –
– +
IP
Input
+ – IgG
– + Flag
CMTM6
p21
HuH-7
HepG2
G
IP
Input
+ –
– +
IP
Input
+ – IgG
– + Flag
CMTM6
p21
HuH-7
HepG2
H
OE-NC
OE-CMTM6
shNC
shCMTM6-1
shCMTM6-2
CMTM6
p21
β-actin
HuH-7
HepG2
I
Vector
CMTM6
0.5
1
2
μg
CMTM6
p21
p53
B-actin
Patient
1 2 3 4 5 6 7 8 9 10 11 12 13
14 15 16 17 18 19 20 21 22 23 24 25 26
CMTM6
p21
β-actin

Figure. 4

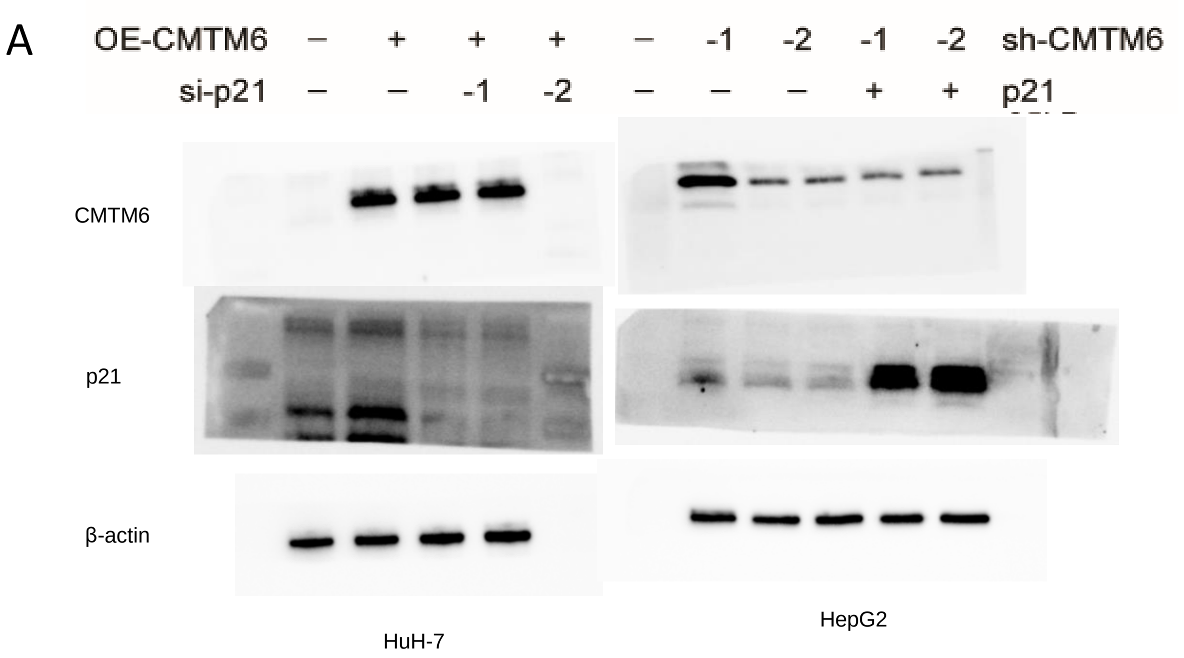
A
OE-CMTM6 – + + + – -1 -2 -1 -2 sh-CMTM6
si-p21 – – -1 -2 – – – + + p21
CMTM6
p21
β-actin
HuH-7
HepG2

Figure. 5

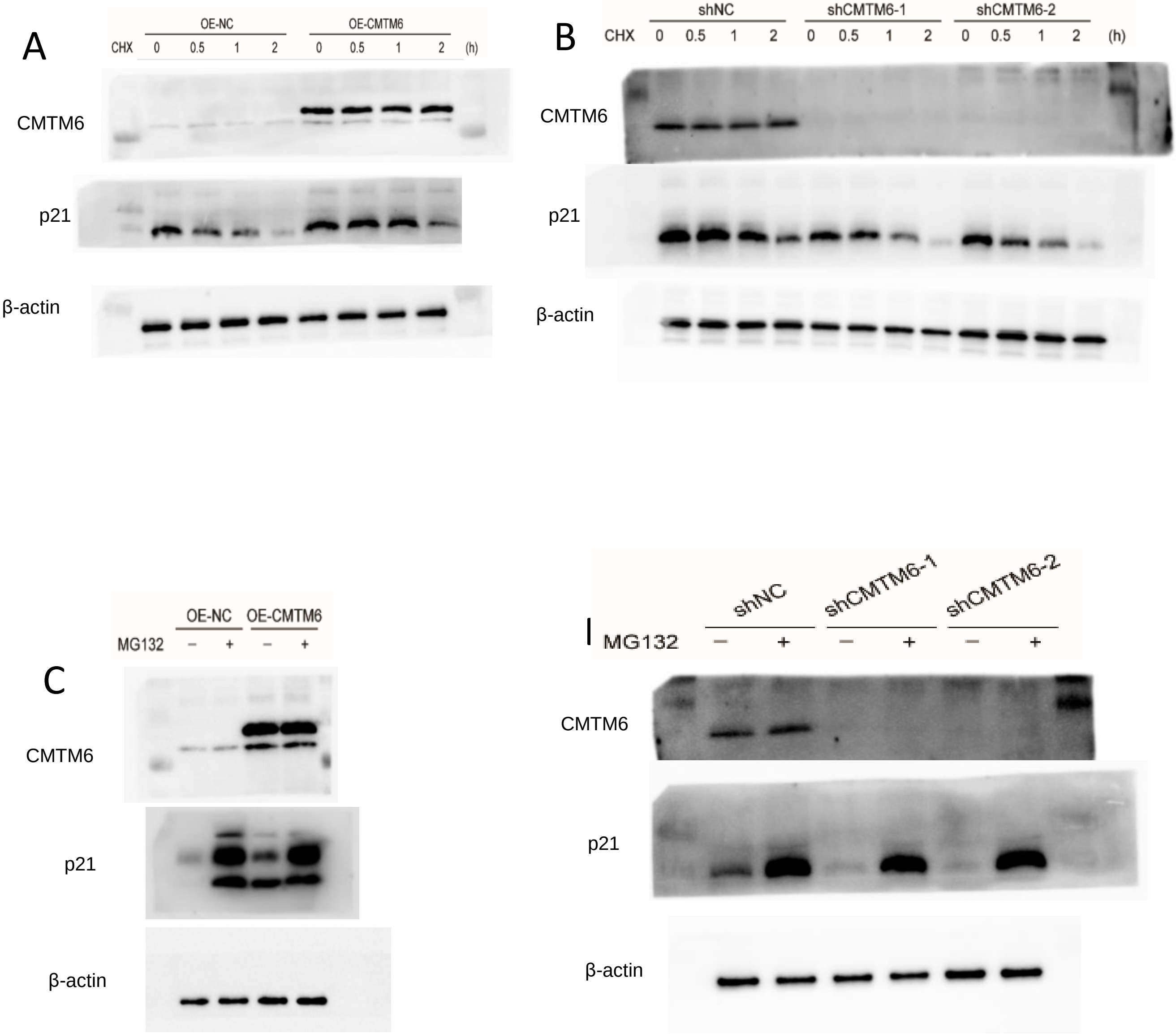
A
OE-NC
OE-CMTM6
CHX 0 0.5 1 2 0 0.5 1 2 (h)
CMTM6
p21
β-actin
B
shNC
shCMTM6-1
shCMTM6-2
CHX 0 0.5 1 2 0 0.5 1 2 0 0.5 1 2 (h)
CMTM6
p21
β-actin
C
OE-NC
OE-CMTM6
MG132 − + − +
CMTM6
p21
β-actin
I
shNC
shCMTM6-1
shCMTM6-2
MG132 − + − + − +
CMTM6
p21
β-actin

Figure. 5

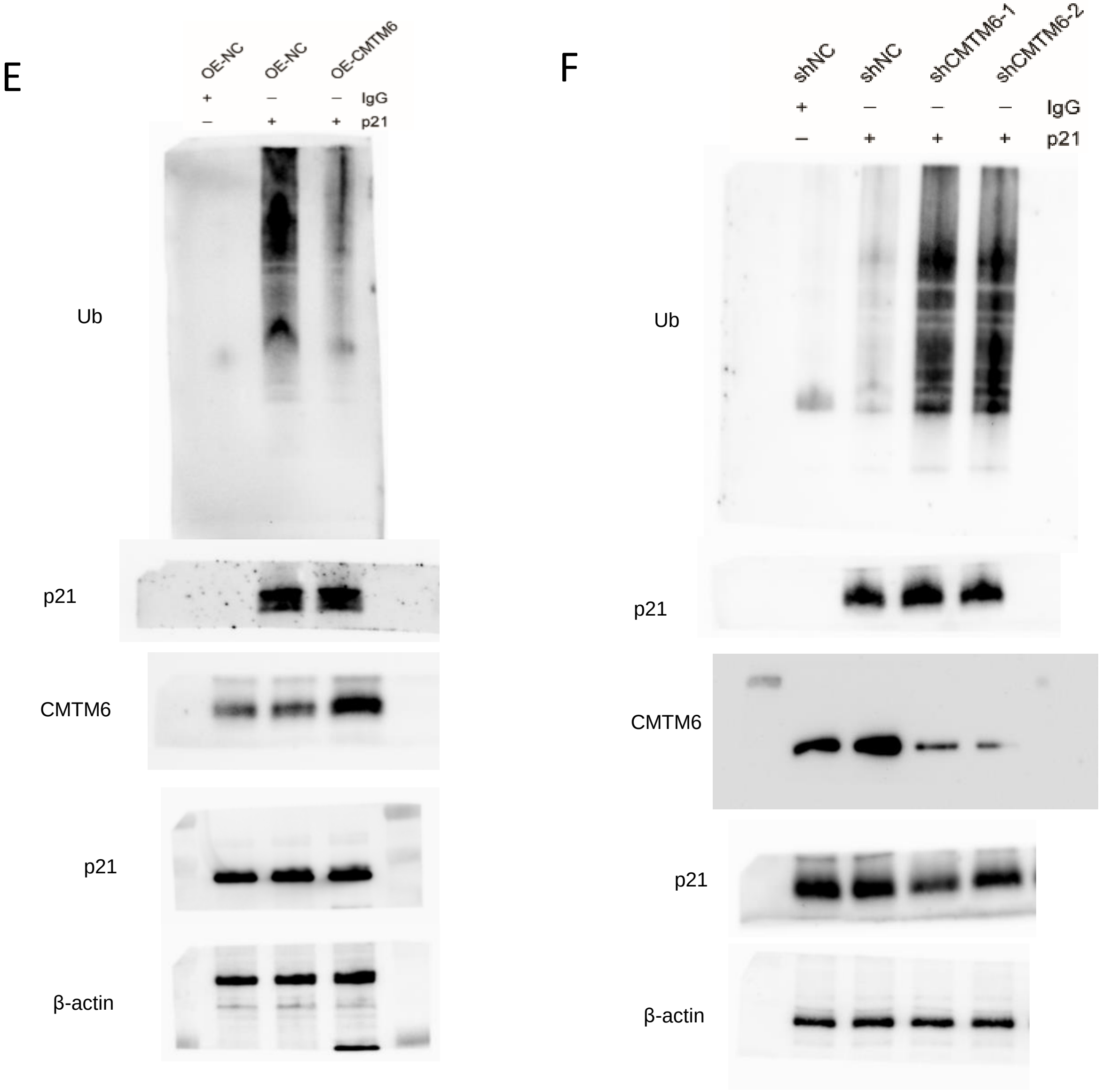
E
OE-NC
OE-NC
OE-CMTM6
+ – – IgG
– + + p21
Ub
p21
CMTM6
p21
β-actin
F
shNC
shNC
shCMTM6-1
shCMTM6-2
+ – – – IgG
– + + + p21
Ub
p21
CMTM6
p21
β-actin

Figure. 5

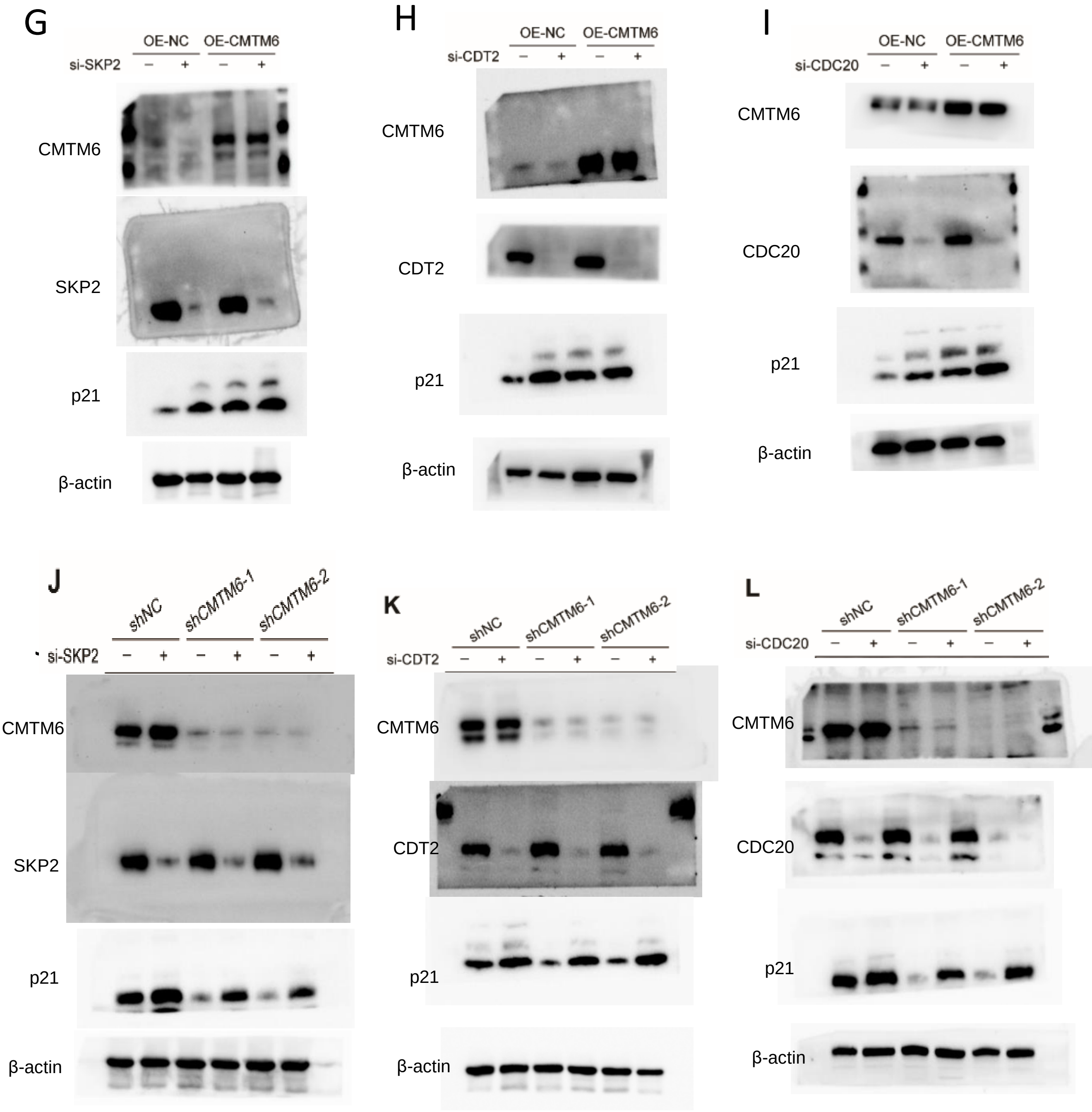

G
OE-NC
OE-CMTM6
si-SKP2
CMTM6
SKP2
p21
β-actin
H
OE-NC
OE-CMTM6
si-CDT2
CMTM6
CDT2
p21
β-actin
I
OE-NC
OE-CMTM6
si-CDC20
CMTM6
CDC20
p21
β-actin
J
shNC
shCMTM6-1
shCMTM6-2
si-SKP2
CMTM6
SKP2
p21
β-actin
K
shNC
shCMTM6-1
shCMTM6-2
si-CDT2
CMTM6
CDT2
p21
β-actin
L
shNC
shCMTM6-1
shCMTM6-2
si-CDC20
CMTM6
CDC20
p21
β-actin

Figure. 6

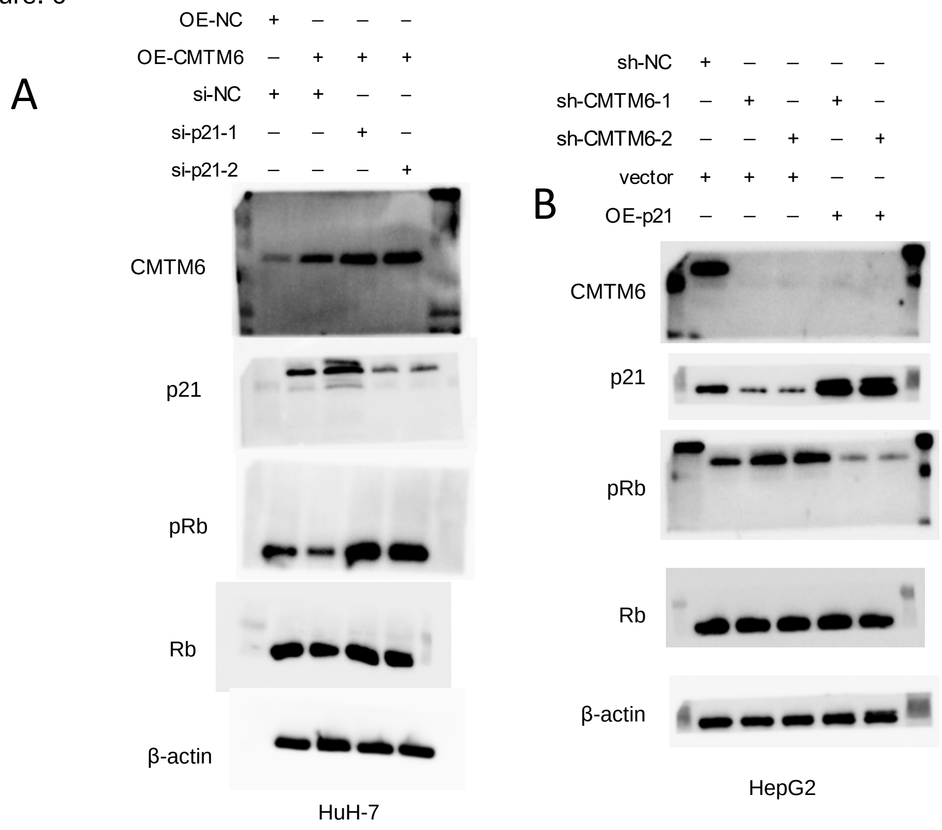

Figure. 7

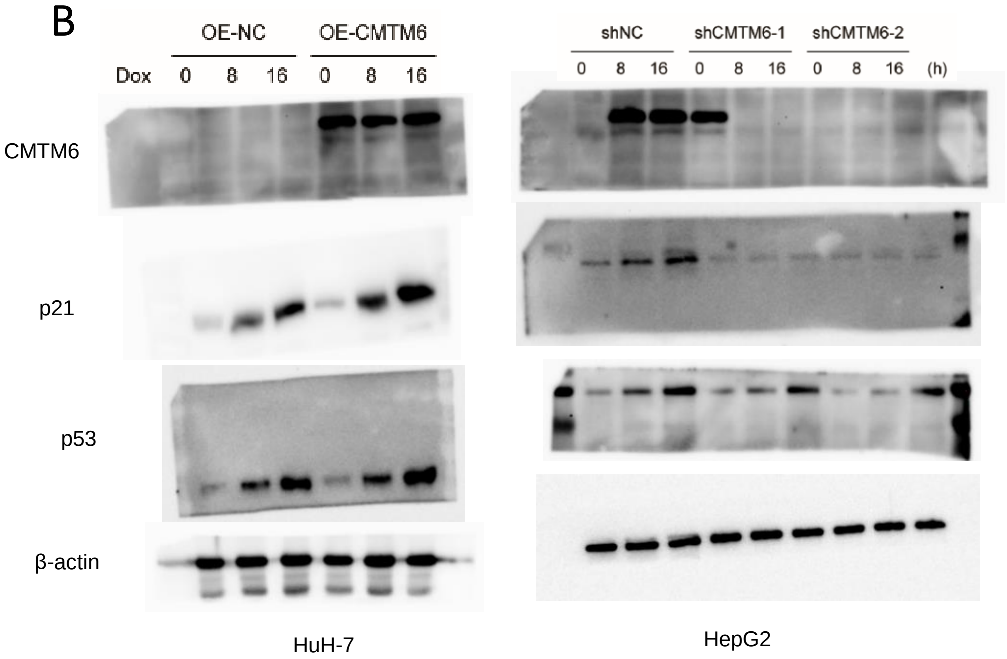

Figure. S1

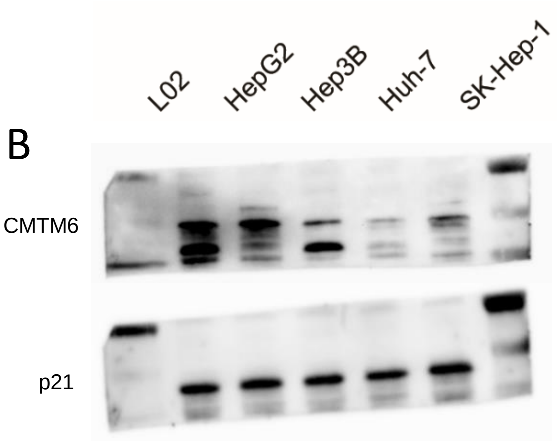

Figure. S2

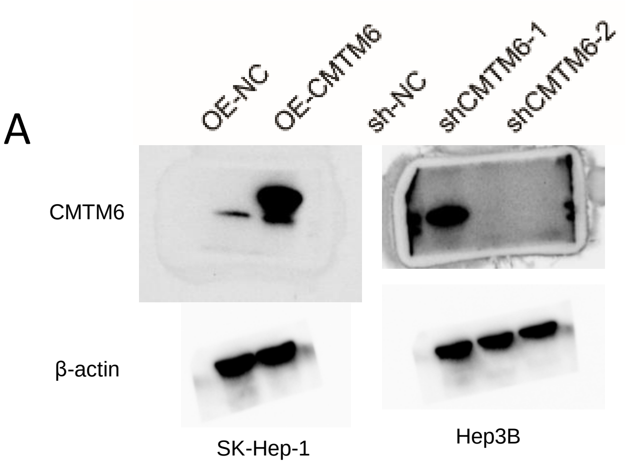

Figure. S5

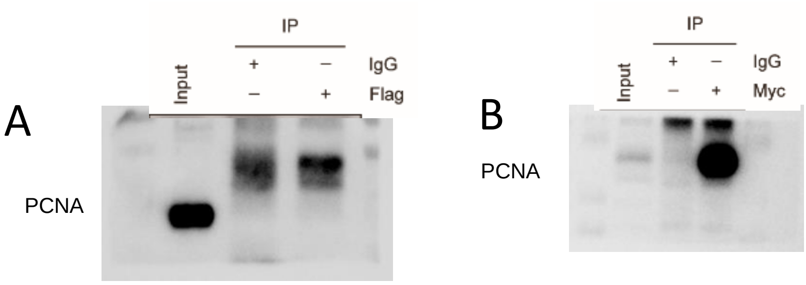

Figure. S6

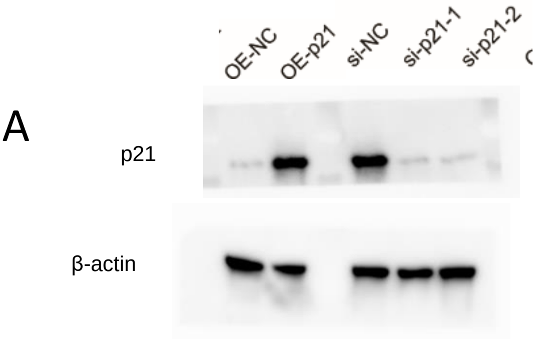

Figure. S7

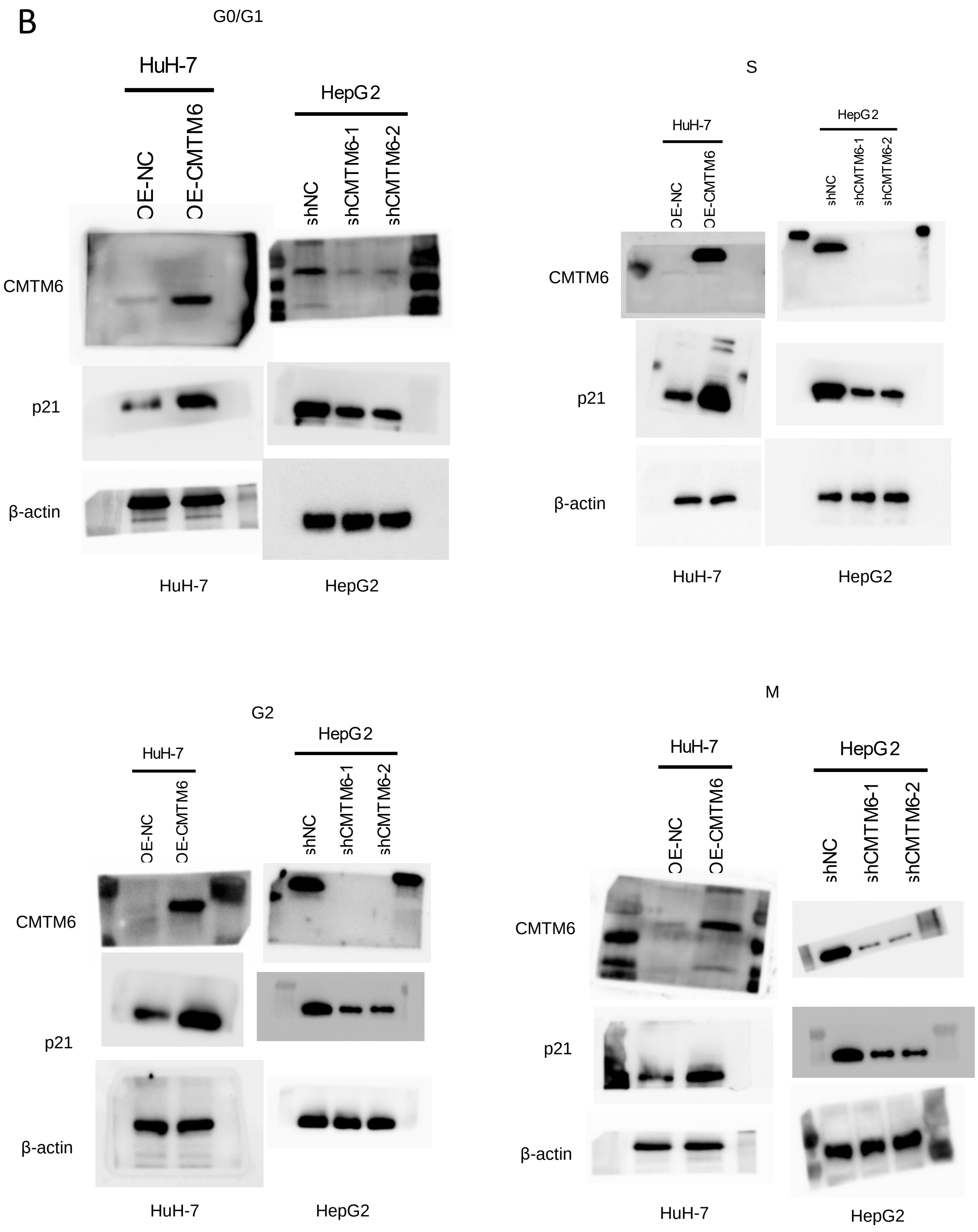
B
G0/G1
HuH-7
OE-NC
OE-CMTM6
HepG2
shNC
shCMTM6-1
shCMTM6-2
CMTM6
p21
β-actin
HuH-7
HepG2
S
HuH-7
OE-NC
OE-CMTM6
HepG2
shNC
shCMTM6-1
shCMTM6-2
CMTM6
p21
β-actin
HuH-7
HepG2
G2
HuH-7
OE-NC
OE-CMTM6
HepG2
shNC
shCMTM6-1
shCMTM6-2
CMTM6
p21
β-actin
HuH-7
HepG2
M
HuH-7
OE-NC
OE-CMTM6
HepG2
shNC
shCMTM6-1
shCMTM6-2
CMTM6
p21
β-actin
HuH-7
HepG2

Figure. S7

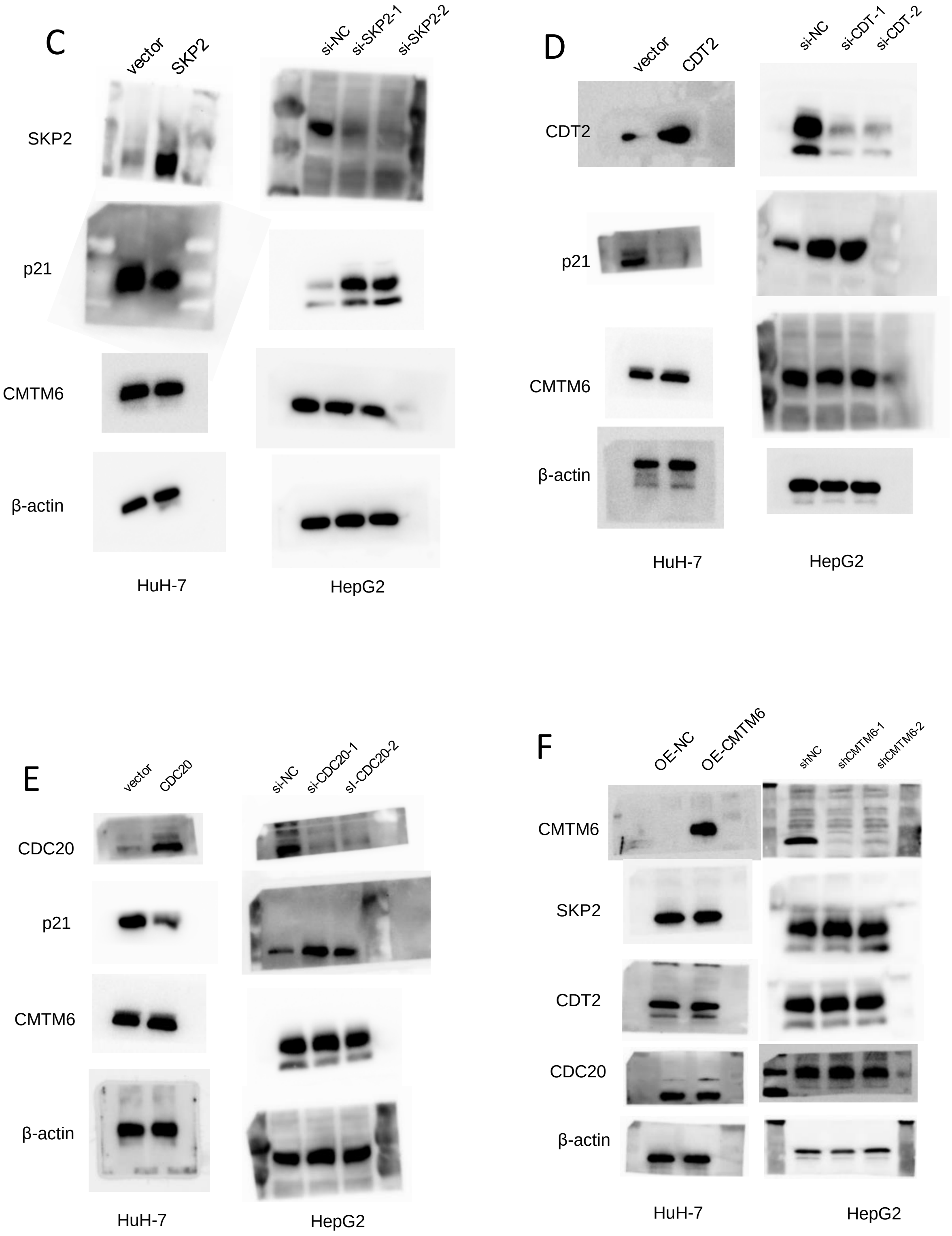
C
vector
SKP2
si-NC
si-SKP2-1
si-SKP2-2
SKP2
p21
CMTM6
β-actin
HuH-7
HepG2
D
vector
CDT2
si-NC
si-CDT-1
si-CDT-2
CDT2
p21
CMTM6
β-actin
HuH-7
HepG2
E
vector
CDC20
si-NC
si-CDC20-1
sI-CDC20-2
CDC20
p21
CMTM6
β-actin
HuH-7
HepG2
F
OE-NC
OE-CMTM6
shNC
shCMTM6-1
shCMTM6-2
CMTM6
SKP2
CDT2
CDC20
β-actin
HuH-7
HepG2

Figure. S7

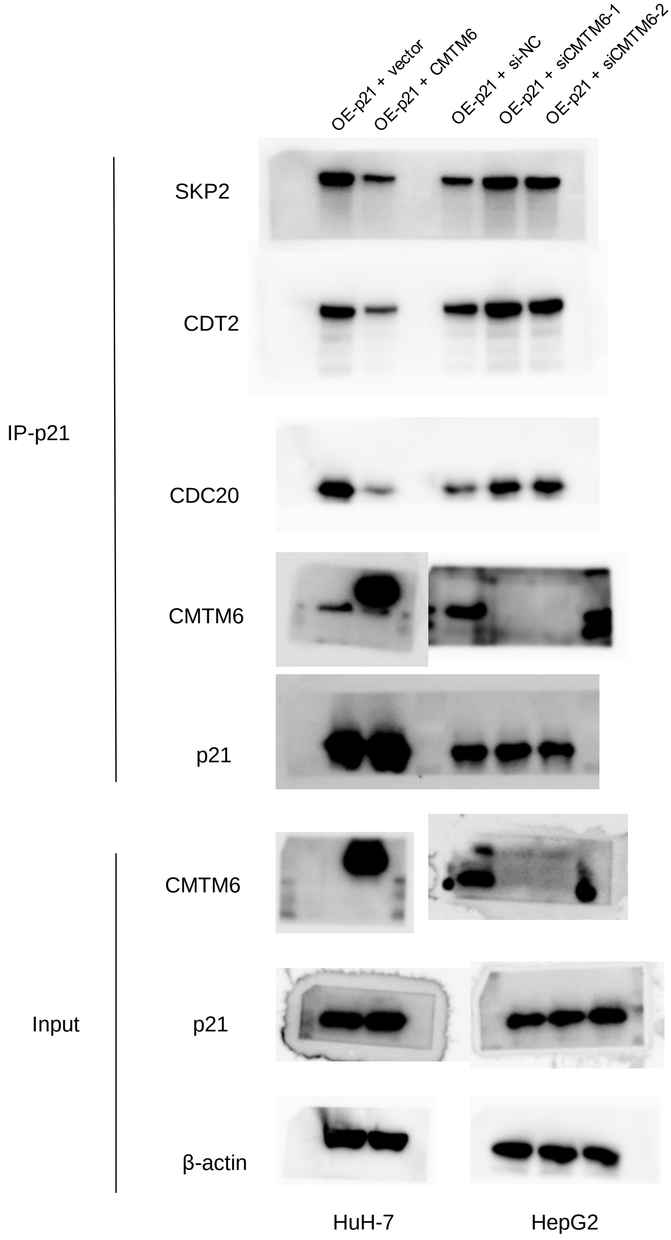
OE-p21 + vector
OE-p21 + CMTM6
OE-p21 + si-NC
OE-p21 + siCMTM6-1
OE-p21 + siCMTM6-2
IP-p21
SKP2
CDT2
CDC20
CMTM6
p21
Input
CMTM6
p21
β-actin
HuH-7
HepG2

Figure. S8

A

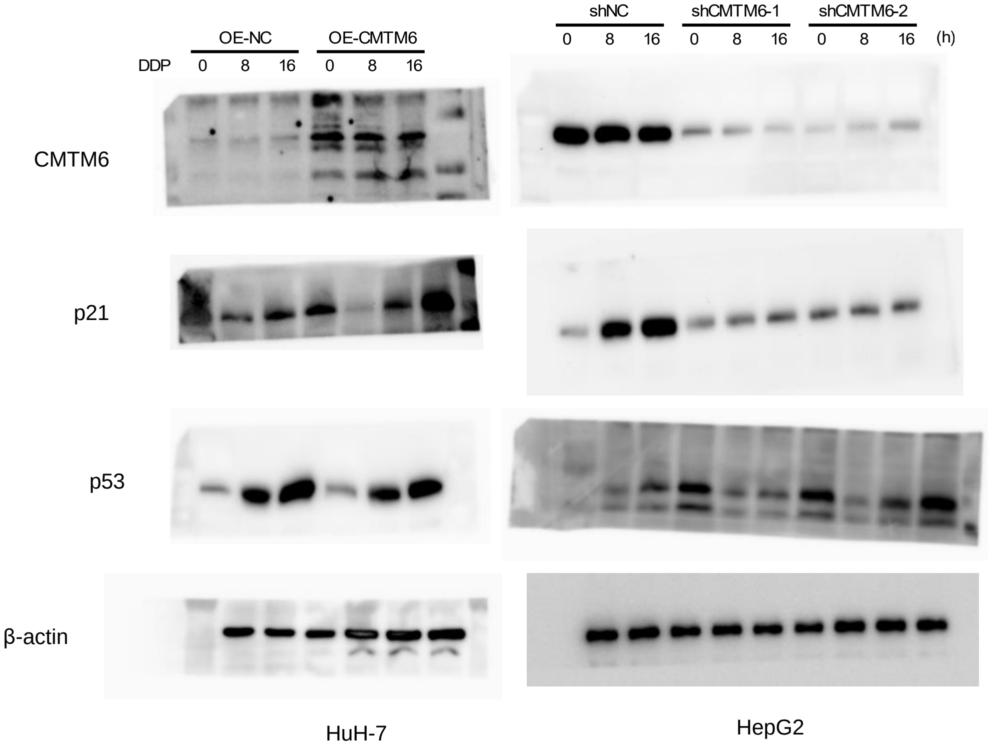
OE-NC
OE-CMTM6
DDP
0
8
16
0
8
16
shNC
shCMTM6-1
shCMTM6-2
0
8
16
0
8
16
0
8
16
(h)
CMTM6
p21
p53
β-actin
HuH-7
HepG2
